# Widespread mRNA Association with Cytoskeletal Motor Proteins and Identification and Dynamics of Myosin-Associated mRNAs in *S. cerevisiae*

**DOI:** 10.1371/journal.pone.0031912

**Published:** 2012-02-16

**Authors:** Jason M. Casolari, Michael A. Thompson, Julia Salzman, Lowry M. Champion, W. E. Moerner, Patrick O. Brown

**Affiliations:** 1 Department of Biochemistry, Stanford University School of Medicine, Stanford, California, United States of America; 2 Howard Hughes Medical Institute, Stanford University School of Medicine, Stanford, California, United States of America; 3 Department of Chemistry, Stanford University, Stanford, California, United States of America; 4 Department of Statistics, Stanford University, Stanford, California, United States of America; University of California San Francisco/Howard Hughes Medical Institute, United States of America

## Abstract

Programmed mRNA localization to specific subcellular compartments for localized translation is a fundamental mechanism of post-transcriptional regulation that affects many, and possibly all, mRNAs in eukaryotes. We describe her e a systematic approach to identify the RNA cargoes associated with the cytoskeletal motor proteins of *Saccharomyces cerevisiae* in combination with live-cell 3D super-localization microscopy of endogenously tagged mRNAs. Our analysis identified widespread association of mRNAs with cytoskeletal motor proteins, including association of Myo3 with mRNAs encoding key regulators of actin branching and endocytosis such as WASP and WIP. Using conventional fluorescence microscopy and expression of MS2-tagged mRNAs from endogenous loci, we observed a strong bias for actin patch nucleator mRNAs to localize to the cell cortex and the actin patch in a Myo3- and F-actin dependent manner. Use of a double-helix point spread function (DH-PSF) microscope allowed super-localization measurements of single mRNPs at a spatial precision of 25 nm in *x* and *y* and 50 nm in *z* in live cells with 50 ms exposure times, allowing quantitative profiling of mRNP dynamics. The actin patch mRNA exhibited distinct and characteristic diffusion coefficients when compared to a control mRNA. In addition, disruption of F-actin significantly expanded the 3D confinement radius of an actin patch nucleator mRNA, providing a quantitative assessment of the contribution of the actin cytoskeleton to mRNP dynamic localization. Our results provide evidence for specific association of mRNAs with cytoskeletal motor proteins in yeast, suggest that different mRNPs have distinct and characteristic dynamics, and lend insight into the mechanism of actin patch nucleator mRNA localization to actin patches.

## Introduction

mRNA localization is a fundamental and widespread mechanism for the post-transcriptional regulation of gene expression across many cell types and species [1], [2]. The localization of messenger ribonucleoprotein (mRNP) complexes to specific subcellular compartments allows for greater spatial and temporal control of gene expression through regulated and localized translation [2], [3]. Indeed, there is increasing evidence that most, if not all, mRNAs are programmed to localize to specific sites in the cell [4], [5]. A systematic study in developing *Drosophila* embryos found evidence of specific subcellular localization for more than 70% of the mRNAs surveyed; in most cases, the localization pattern paralleled that of the encoded proteins [4]. The dynamic localization of mRNAs to discrete cytological locations is critical for such diverse processes as asymmetric cell fate determination, cell motility, and the establishment of cellular and organismal polarity [1], [2].

Among the most fully understood examples of dynamic mRNA localization is the transport of the *ASH1* mRNA to the bud tip of *Saccharomyces cerevisiae* to impart asymmetric production of a transcriptional regulator that effects cell fate determination [6], [7], [8], [9]. The *ASH1* transcript is transported by the type V myosin, Myo4, and other components of the “locasome,” an mRNP complex that also includes the RNA binding protein (RBP), She2, which recognizes the localization elements in the *ASH1* transcript, as well as She3, an adapter protein that bridges the interaction between She2 with Myo4 [8], [9], [10], [11], [12], [13] and which also directly binds RNA [14]. In order to directly observe *ASH1* dynamics, Bertrand et al. [9] constructed a chimeric mRNA comprised of a reporter gene, the *ASH1* 3′-UTR, and many copies of a viral (MS2) RNA hairpin that is capable of binding the MS2 viral coat protein (MS2-CP) with nanomolar affinity [15], [16], [17]. When the MS2-CP was fused to GFP and expressed in cells harboring the *ASH1* chimeric transcript, Bertrand et al. were able to visualize the dynamics of the mRNP in live cells [9]. Subsequent genome-wide studies have indicated that more than 20 transcripts associate with Myo4 and translocate from the mother cell to the budding daughter cell tip, where they, along with *ASH1*, are believed to be anchored in an actin-dependent manner [6], [7], [8], [13], [18], [19].

Actin-dependent mRNA localization appears to be widespread [1], [2], [3]. One of the best-studied cases is the mRNA encoding β-actin itself. *β-actin* mRNA localizes to lamellapodia at the leading edge of crawling chick and mammalian fibroblasts [20], [21], [22]; its localization is required for the enhancement of cytoskeletal polarization, cell motility and directionality in crawling [23], [24]. Localization of *β-actin* mRNA correlates with locally elevated levels of β-actin protein, supporting the model that transcript localization facilitates localized translation of *β-actin* [23]. As actin is highly dynamic, localized production of the β-actin monomer would increase the local concentration of the β-actin pool to facilitate the rapid polymerization necessary for cytoskeletal rearrangement and cell movement. Further studies have indicated that the seven mRNAs that encode the Arp2/3 subunits, which are necessary for actin branching, are also localized to the leading edge of fibroblasts [25]. From the studies of *β-actin* and *Arp2/3* mRNA localization, an interesting model has emerged in which cells reinforce polarization by transporting mRNAs encoding proteins that modify cytoskeletal polarization along the cytoskeleton itself [23], [25].

Microtubules and the associated dynein and kinesin motor proteins are also implicated in dynamic mRNA localization in higher organisms [1], [2], [3]. For example, both *bicoid* and *gurken* mRNAs have been shown to undergo dynein-dependent localization in *Drosophila* oocytes [26], [27], [28], [29]. Interestingly, the dynein motor that facilitates movement of the *gurken* mRNA to the anterior of the oocyte serves as an anchor for the transcript once it is properly localized, indicating that motor proteins may play multiple roles in dynamic mRNA localization [30], [31].

Several mRNAs have been found to have kinesin-dependent localization, including *Vg1* mRNA in *Xenopus* oocytes [32], [33] and *oskar* mRNA in *Drosophila* oocytes [34], *CaMKIIα* in mammalian neurons [35], and *MBP* in oligodendrocytes [36]. *Vg1* mRNA, which encodes a member of the transforming growth factor β family, is localized to the vegetal pole of *Xenopus laevis* oocytes [37]; its localization depends on two different kinesins in a portion of the oocyte with mixed microtubule orientations [38]. Studies of *oskar* mRNA dynamics in live *Drosophila* oocytes, where Oskar localization to the posterior pole is important for body axis patterning [3], have revealed a similarly complex localization mechanism. Quantification of *oskar* dynamics in live cells has suggested that *oskar* mRNA is able to move in a kinesin-dependent manner in all directions along a mixed-polarization microtubule cytoskeleton with a weak overall bias towards the posterior [39].

As evidenced in the studies of *Vg1*, *oskar*, and *ASH1*, careful measurement of mRNP dynamics has been critical for revealing the mechanisms that impart asymmetric sorting. It is clear that various proteins and signaling events alter the composition and presumably the dynamics of the *ASH1* mRNP between its birth in the nucleus and its ultimate localization in the cell. Recent studies of *ASH1* localization have revealed that the She2 protein is recruited to the mRNA co-transcriptionally and is believed to accompany the mRNA into the cytoplasm, where it encounters Myo4 for eventual translocation to the bud tip [40]. Another RBP, Puf6, serves as a translational repressor, believed to associate with the mRNA until the *ASH1* transcript is released from repression at the bud tip [41], [42]. Further studies have demonstrated that paralogous *S. cerevisiae* ribosomal proteins, previously thought to be functionally interchangeable, differentially affect the ability of *ASH1* to be properly localized [43]. This finding suggests the intriguing possibility that different classes of ribosomes themselves may differentially associate with specific mRNAs, providing an additional mechanism for regulation of mRNA localization.

While most studies of mRNA dynamics in *S. cerevisiae* have focused on *ASH1* and other transcripts associated with Myo4, we hypothesized that other cytoskeletal motor proteins might have roles in mRNA localization in yeast. In order to test this hypothesis, we immunopurified the five actomyosin motors, Myo1, Myo2, Myo3, Myo4, and Myo5; the kinesin-like proteins Kar3, Kip1, Kip2, Kip3, and Smy1; and the dynein, Dyn1, from *S. cerevisiae* and profiled associated RNAs using DNA microarrays. This analysis established that six of the eleven cytoskeletal motor proteins associate with functionally specific sets of mRNAs, indicating that asymmetric localization of mRNAs via association with motors is widespread. Among the new mRNA interactions identified is the association of Myo3 with mRNAs encoding key regulators of endocytosis and actin patch dynamics. We used conventional fluorescence microscopy and a microscope exhibiting a double-helix point spread function (DH-PSF) for 3D super-localization microscopy of MS2-GFP-tagged mRNAs to quantitatively profile the localization and dynamics of a Myo3-associated actin patch mRNA at high temporal and spatial precision in three dimensions. Tracking of single actin patch-localized mRNPs with an average spatial precision of 25 nm in *x* and *y* and 50 nm in *z* allowed us to derive 3D diffusion coefficients and measures of confinement, as well as quantitative measurements of the contribution of Myo3 and filamentous actin (F-actin) to actin patch mRNA dynamics, providing insight into the mechanism of actin patch (AP) mRNA localization to actin patches in live cells.

## Results

### Immunopurification of Cytoskeletal Motor Proteins

**Figure 1:**
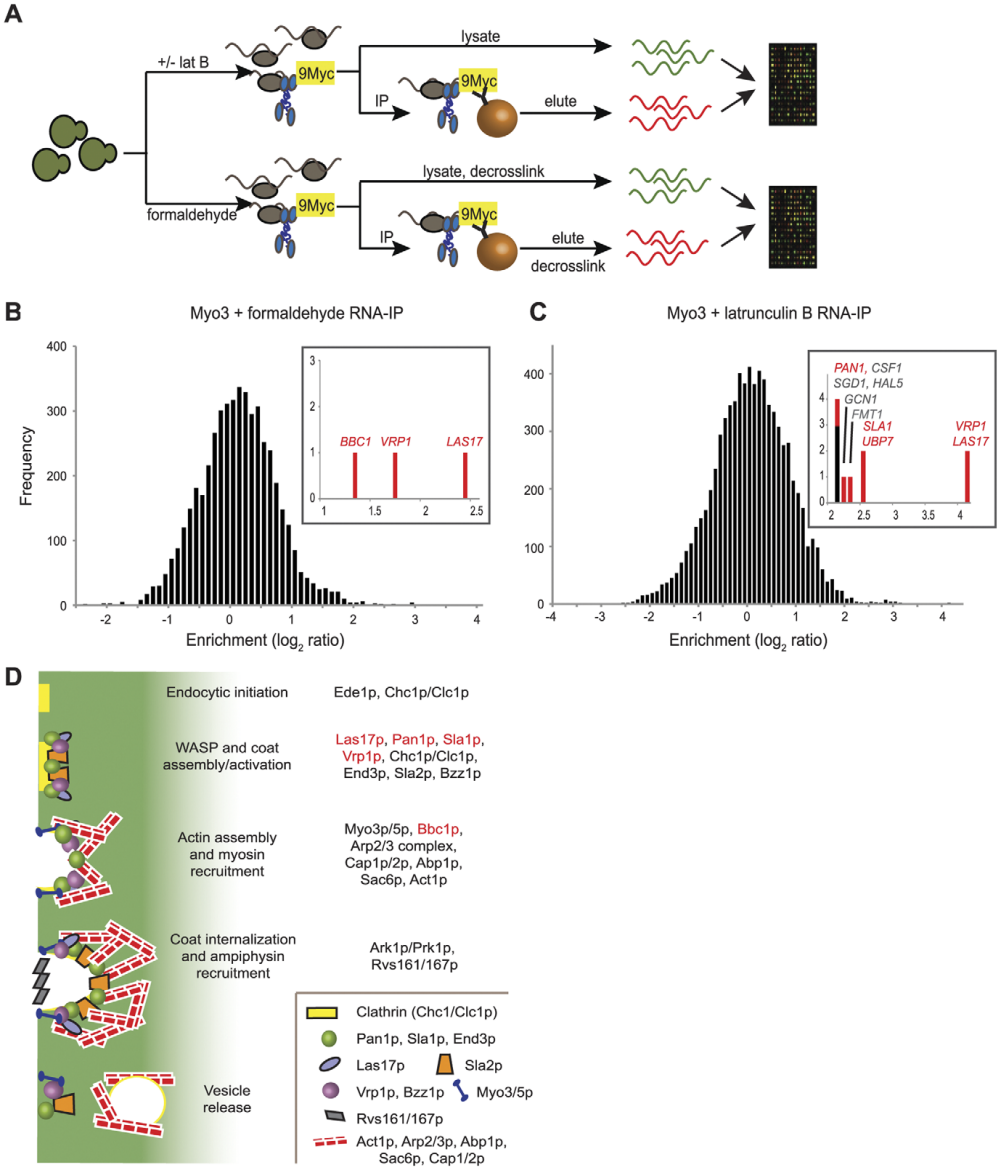
Myo3 associates with RNAs encoding key regulators of actin nucleation at actin patches. (A) Identification of mRNAs associated with motor proteins in *S. cerevisiae*. Cells encoding motor proteins C-terminally tagged with 9Myc (shown) or EGFP were treated with latrunculin B (top) or fixed with formaldehyde (bottom). Cells were lysed and the motor proteins associated with mRNA cargoes were immunopurified using antibodies coupled to magnetic beads. Enriched mRNAs were eluted from the magnetic beads and, in the case of formaldehyde treatment, decrosslinked before labeling with cyanine dyes. Enriched mRNAs and mRNAs extracted from lysate were comparatively hybridized to yeast DNA oligonucleotide microarrays. (B) and (C) Distribution of the average log_2_ Cy5/Cy3 fluorescence ratios from four independent IPs and microarray hybridizations for identification of mRNAs associated with Myo3p in the presence of formaldehyde crosslinker (B) or after cells were incubated with latrunculin B (C). The insets show the mRNAs significantly enriched in Myo3p IPs after subtraction of mRNAs found in mock IPs. mRNAs encoding factors involved in endocytosis at actin patches are labeled in red. (D) Schematic of the steps of endocytic internalization at actin patches and the relative timing of arrival of proteins involved in the regulation and formation of the endosome. Proteins encoded by mRNAs associated with Myo3p are colored in red as in parts (B) and (C).

To enable efficient immunopurification (IP) of cytoskeletal motor proteins in *S. cerevisiae*, we constructed strains in which nine Myc epitopes were fused to the C-terminus of each of six cytoskeletal motor proteins by knock-in at the endogenous loci ([44]; Materials and Methods). For some studies, we used strains in which a C-terminal enhanced green fluorescent protein (EGFP) was fused to selected motor proteins ([45]; Table S1). We used formaldehyde crosslinking to stabilize associations between motors proteins and interacting RNAs before IP. Yeast cells growing exponentially in rich medium were treated with formaldehyde, lysed and sonicated, then incubated with magnetic beads coupled to monoclonal antibodies against either 9Myc or GFP (Figure 1A) to isolate the tagged motor proteins along with any associated RNAs. After IP, the formaldehyde crosslinks were reversed and the enriched RNAs and RNAs purified from the corresponding total lysate were amplified and labeled respectively with Cy5 and Cy3, then jointly hybridized to custom-made, *S. cerevisiae* oligonucleotide microarrays [46].

Several controls for the IPs were performed. Out of concern that formaldehyde might stabilize spurious association of mRNA with the motor proteins, we attempted alternative purification approaches that did not involve crosslinking. While soluble motor proteins could be immunopurified in the absence of formaldehyde, their specific association with mRNAs was less consistent in the absence of crosslinking, and much of the motor protein was found to partition to the insoluble fraction (data not shown). The addition of a low concentration of latrunculin B (2 µg/ml) to live cells to partially solubilize the actin cytoskeleton [47] allowed for successful IP of the motor proteins and associated mRNAs without the need for a chemical crosslinker (Figure 1A). In both the case of latrunculin B and formaldehyde treatment, we also performed IPs in which the untagged parent yeast strains were processed for IP and microarray analysis in a manner identical to that of the tagged strains.

We used the significance analysis of microarrays (SAM) algorithm [48] to identify mRNAs that were specifically enriched in association with a tagged motor protein and not in the mock IPs, as well as to estimate a corresponding false discovery rate (FDR). Using these methods for Myo4, which served as our positive control, we identified most of the RNAs previously reported as Myo4 mRNA targets with both the formaldehyde crosslinking and latrunculin B procedures at an FDR of less than 5% ([18]; Dataset S1).

With the same procedures, we identified mRNAs specifically associated with Myo1, a type II myosin motor that localizes to the actomyosin ring at the bud neck and is required for proper cytokinesis and cell separation [49], [50], [51]. Using both the formaldehyde crosslinking and latrunculin B procedures, Myo1 associated with no mRNAs under a local FDR cutoff of 5%. However, Gene Ontology (GO) analysis [52], [53], [54] of the top 465 Myo1-associated mRNAs (global FDR<7%) upon treatment with latrunculin B revealed that the “protein kinase activity,” “cellular bud neck,” and “site of polarized growth” categories are specifically enriched, including the mRNAs *BUD4*, *IQG1*, *BOI2*, *NIS1*, and *SWE1*, among others, whose protein products localize to the bud neck (Dataset S1; [55], [56], [57], [58], [59]). Finding Myo1 associated with mRNAs encoding regulators of cytokinesis and budding suggests that perhaps these mRNAs co-localize with Myo1 at the bud neck for localized protein production and regulation of cell separation.

We identified several mRNAs specifically and reproducibly associated with Myo2 (Dataset S1), a type V actomyosin motor that has been previously reported to travel on actin cables and associate with a variety of organelles such as mitochondria [60], [61], [62], vacuoles [63], [64], peroxisomes [65], [66], and vesicles [67] for the establishment of polarity and the delivery of factors into the emerging bud. In contrast to previous reports which identified more than 2,300 Myo2-associated mRNAs [68], we found far fewer mRNAs significantly associated (FDR<5%) with Myo2; in IPs from Myo2:9Myc tagged cells treated with latrunculin B, we found 429 mRNAs, while IPs of Myo2:9Myc and Myo2:GFP from crosslinked cells yielded only 58 and 12 mRNAs, respectively. Intriguingly, among the Myo2-associated mRNAs were several previously found to associate with Myo4, including *ASH1*, *IST2*, *TCB3*, and *CLB2* [13], [18]. In addition, we found mRNAs enriched in the GO categories of “actin cytoskeleton organization” and “maintenance of cellular polarity,” including *WSC2*, *BNI1*, *SLA1*, *CAP2*, *SPA2*, *BUD8*, and *BUD9*. Furthermore, Myo2 was selectively associated with mRNAs encoding components of organelle membranes, including Mmr1, Vac8, and Vac17, which have been previously reported to facilitate Myo2 localization of bud-localized mitochondria and the vacuole, respectively [61], [69], [70].

The kinesin-like proteins Kip1, Kip2, and Smy1 [71], and the dynein protein, Dyn1, did not reproducibly associate with any mRNAs in our assays (Dataset S2). The kinesin-8 type Kip3 protein, however, associated with 327 mRNAs at <5% FDR, among which mRNAs with roles in mitochondria were enriched. While Kip3 has not been linked to mitochondria in *S. cerevisiae*, a kinesin-8 type motor has been found associated with mitochondria in *Drosophila* [72], suggesting the possibility for further Kip3 roles in budding yeast. The Kar3 kinesin-like motor, which is required for nuclear fusion during mating [73], was selectively associated with mRNAs encoding factors that play roles in conjugation and cellular fusion including Cdc12, a septin ([74]; Dataset S2).

### Myo3 Associates with mRNAs Encoding Actin Patch Nucleating Proteins

Myo3, a type I actomyosin motor that, like Myo5, localizes to actin patches at the cortex and plays a role in actin nucleation and the regulation of endocytosis [75], [76], [77], [78], [79], reproducibly associated with a specific set of mRNAs encoding key regulators of actin branching and the endocytic process. The mRNAs *BBC1*, *VRP1*, and *LAS17* were specifically enriched with Myo3 after formaldehyde crosslinking (Figure 1B, inset; Dataset S1). *LAS17* encodes the yeast homolog of WASP, which functions as a nucleation promoting factor (NPF) that interacts with type I myosins [14], [77] and activates the Arp2/3 complex to facilitate actin branching at the site of endocytosis [80]. Vrp1, otherwise known as WIP, also serves to stimulate the NPF activity of the type I myosin, Myo5 [79]. Bbc1 is recruited to actin patches with Myo5 and has been suggested to regulate the activity of both Myo5 and Las17 [79], [81].

Immunopurification of Myo3 from non-crosslinked cells pre-incubated with latrunculin B also selectively enriched *VRP1* and *LAS17* mRNAs (Figure 1C, inset; Dataset S1) along with three other mRNAs encoding factors that regulate actin patch nucleation: *UBP7*, *SLA1*, and *PAN1*. Sla1 binds to the Arp2/3 regulator, Pan1, and Ubp7 is a ubiquitin-specific protease that has previously been shown to interact with Myo3 [82], [83], [84]. Additional mRNAs that consistently co-purified with Myo3 in the presence of latrunculin B include RNAs encoding Csf1, a protein localized to mitochondria [85]; Sgd1, a nuclear protein involved in the osmoregulatory response to glycerol [86]; Hal5, a putative protein kinase involved in halide tolerance [87]; Gcn1, involved in translational control [88], [89]; and Fmt1, which catalyzes initiator-Met-tRNA formylation in mitochondria [90].

We focused our subsequent investigations on the Myo3-associated mRNAs for a number of reasons. First, actin patches are known to be dynamic structures [91], [92] whose regulation is tightly controlled through the sequential recruitment of a series of proteins (Figure 1D; [79], [81], [93]). Thus, dynamic localization of mRNAs to these structures could play a role in the localized production of proteins to aid in the regulation of endocytosis and budding. Second, Myo3-associated mRNAs encode the key regulators of the actin branching process crucial for successful invagination and release of endosomes from the cell cortex [79], [81], [93], [94], [95]. However, Myo3 does not appear to associate with every mRNA that encodes a protein known to interact at the actin patch (Figure 1D; Dataset S1 and Table S2). The other type I myosin present at actin patches, Myo5, associated with the actin patch (AP)-regulating mRNAs *SLA1* and *VRP1* with a FDR of 23% upon treatment with latrunculin B (Dataset S1), reflecting a less significant and reproducible interaction with the AP mRNAs. Additional IPs using the same procedure with genetically tagged Las17 failed to enrich for the AP RNAs that associate with Myo3 (Dataset S1); Vrp1 was associated with its own transcript upon crosslinking, but did not significantly associate with other AP-regulating mRNAs. The weak or absent association of Myo5, Las17, and Vrp1 with AP mRNAs, as well as the presence of EDTA in our purification buffers (see Materials and Methods), which would disrupt any polysomal mRNAs that might interact with Myo3 indirectly through actin patch-localized ribosomes, suggests that Myo3's association with key AP mRNAs is specific. Third, there is precedent for the localization of *β-actin* mRNA and mRNAs encoding the seven Arp2/3 components to sites of actin branching and cytoskeletal rearrangement in mammalian cells [20], [21], [22], [23], [24], [25]. Because actin patches are focal points of cytoskeletal polarity in *S. cerevisiae*, our result suggests that Myo3 association with AP mRNAs may reflect a common mechanism for localization of mRNAs encoding actin cytoskeleton polarization via association with the actin cytoskeleton itself.

### Myo3-Associated mRNAs Localize to Actin Patches

**Figure 2:**
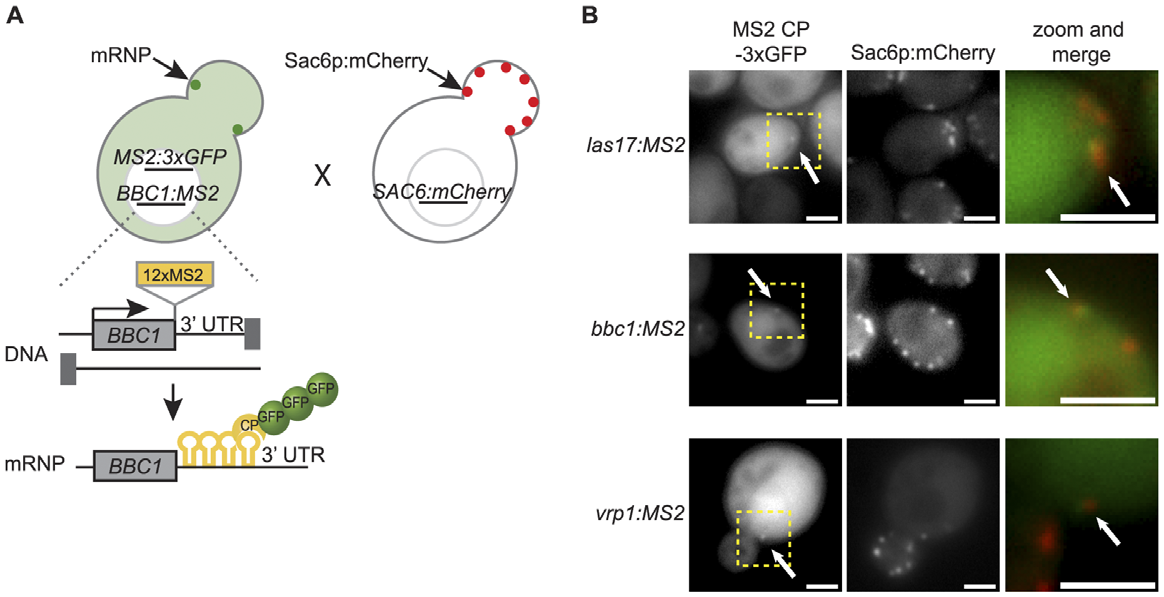
Actin patch nucleator mRNAs are localized to actin patches. (A) Schematic of fluorescent strain construction for visualization of mRNA particles and actin patches in live cells. The sequence for 12 MS2 hairpins was integrated between the coding region of the gene of interest (e.g. *BBC1*) and the 3′ UTR at the native genomic locus. Cells with the gene:MS2 construct also harbored a plasmid allowing inducible expression of the MS2 hairpin-binding coat protein (CP) fused to three tandem copies of enhanced green fluorescent protein (3xGFP), allowing visualization of discrete mRNA particles in live cells. Haploid strains harboring the MS2 constructs were mated to haploid strains harboring an integrated mCherry C-terminal fusion of the *S. cerevisiae* fimbrin protein, Sac6p. (B) Visualization of MS2-CP-3xGFP tagged mRNAs (left panel) and Sac6p:mCherry (middle panel) in diploid cells. Areas of interest in the left panel, highlighted by the yellow bounding box, are shown in the right panel. mRNAs of interest are highlighted by a white arrow. Scale bars are 2 µm.

To test the hypothesis that the Myo3-associated AP mRNAs would localize to actin patches, we used a modified MS2-tagging system that allows expression of the mRNA from its native promoter and preserves the 3′ UTR sequence [96]. We integrated sequences encoding a series of twelve MS2 coat protein binding RNA hairpins between the respective termination codons of *BBC1*, *LAS17*, and *VRP1* and their respective 3′-UTR sequences in separate strains (Figure 2A and Materials and Methods). In each of these strains we expressed an MS2 coat protein (MS2-CP) lacking a nuclear localization sequence and fused to three tandem copies of EGFP under the control of a methionine-inducible promoter [8], [96]. Upon induction, the MS2-CP-3xEGFP protein accumulates in the cytoplasm, but is selectively enriched at the site of the MS2 hairpin-tagged mRNAs, allowing for visualization of the corresponding mRNPs as distinct, bright foci [96]. Induction of the MS2-CP-3xGFP in a control strain lacking any MS2 hairpin RNAs led to bright foci in only 0.01% of cells (data not shown) and their morphologies were distinct from those representing *bona fide* mRNP complexes.

AP-MS2 mRNA haploid strains were mated to a strain in which the Sac6 fimbrin, a marker of actin patches [79], [81], [97], was genetically tagged at its C-terminus with the mCherry protein (Figure 2A). As displayed in Figure 2B, the AP mRNA GFP signal of the *LAS17*, *BBC1*, and *VRP1* mRNPs co-localizes with Sac6 mCherry signal in images taken from live cells, indicating that the AP mRNPs localize to actin patches. In order to quantify the coincidence of AP mRNPs at actin patches, we examined thousands of two-color images of live cells harboring AP mRNPs or two control mRNAs, *ARG3* and *ASH1*. The *ARG3* mRNA, which encodes ornithine carbamoyltransferase in *S. cerevisiae*, was chosen because it was not bound by Myo3 in the presence of formaldehyde or latrunculin B (Dataset S1), is expressed at just 1–2 mRNA copies per cell, similar to the AP mRNAs [98], [99], and because it encodes a cytoplasmic enzyme that is not known to exhibit asymmetric localization [45], [100], [101]. *ASH1* was chosen as a putative positive control; while *ASH1* mRNA was not found associated with Myo3, previous studies have indicated that *ASH1* may anchor at cortical actin in the bud tip [7], [8].

### Deletion of *MYO3* and Disruption of Actin Impair Localization of Actin Patch mRNAs to Actin Patches

**Figure 3:**
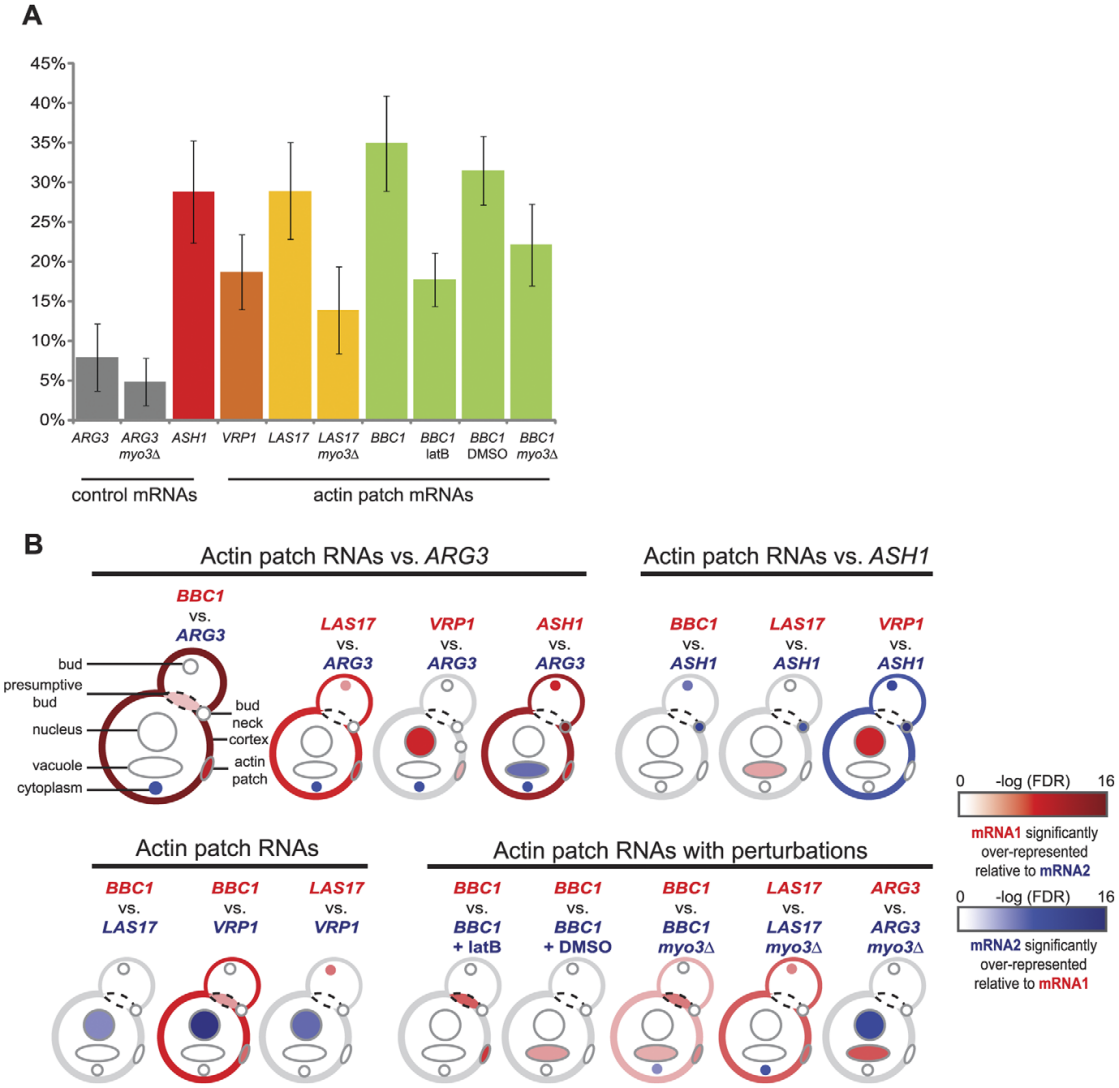
Quantification of actin patch nucleator mRNA localization. (A) Proportion of total mRNAs localizing to the actin patch from cells at all stages of the cell cycle. Error bars represent the 95% confidence interval. (B) Comparison of mRNA localization patterns for pairs of MS2-tagged mRNAs. Cellular structures scored for mRNA localization are indicated in the first ideogram. Significant differences in localization between mRNAs of interest to cellular locations in the ideograms are indicated by color: red or blue indicates a –log false discovery rate (FDR) of 2 (light color) to 16 (saturated, dark color), with red colors indicating significant over-representation of mRNA 1 in each pair and blue colors indicating significant over-representation of mRNA 2. Grey or white represents no significant difference in the localization between the two mRNAs. Significance was measured by Fisher's exact test with the Benjamini-Hochberg multiple hypothesis test correction.

As shown in Figure 3A, we observed significant differences among *VRP1*, *LAS17*, *BBC1*, and *ASH1* in the proportion of mRNAs localized to actin patches (19%, 29%, 35%, and 29%, respectively) when compared to the control mRNA, *ARG3* (8%). Pairwise differences in the proportion between *ARG3* and each of *VRP1*, *LAS17*, *BBC1*, and *ASH1* were each significant by Fisher's exact test (*P* = 3.7×10^−4^, *P* = 3.9×10^−8^, *P* = 2.2×10^−12^, and *P* = 1.9×10^−8^, respectively; Table S3). Addition of latruculin B, which destabilizes F-actin, also led to a significant decrease in the proportion of *BBC1* mRNA co-localizing at actin patches (*P* = 1.6×10^−7^), while addition of the drug carrier, DMSO, had no significant effect (*P* = 0.46).

To test the importance of Myo3 in AP mRNA localization to actin patches, we constructed homozygous *MYO3* null (*myo3*Δ) diploid versions of the *BBC1:MS2 SAC6:mCherry*, *LAS17:MS2 SAC6:mCherry*, and *ARG3:MS2 SAC6:mCherry* strains (see Materials and Methods). Loss of *MYO3* significantly decreased the proportion of *BBC1* and *LAS17* mRNAs that localized to the actin patch (22% and 14%, respectively) while having no significant effect on the localization of *ARG3* (5%; *P* = 5.6×10^−5^, *P* = 1.1×10^−5^, and *P* = 0.50, respectively). *MYO3* deletion does not impact the formation of actin patches [76], nor did the strains exhibit any obvious defects in growth rate, morphology, or abundance of actin patches (data not shown), suggesting that Myo3 selectively promotes the transport of AP mRNAs to actin patches, their retention at actin patches, or both.

### Actin Patch mRNA Localization Patterns are Distinct from Those of *ASH1* and *ARG3* mRNAs

Expression of the MS2-GFP protein in the cytoplasm of AP-MS2 cells had the incidental benefit of demarcating several cytological features; the GFP signal is excluded from the vacuole [8] and slightly enriched in the nucleus. Since the cell cycle phase of *S. cerevisiae* cells can be inferred from bud size, nuclear positioning, and the asymmetric localization of actin patches, we carried out a careful examination of AP mRNA localization across all stages of the cell cycle and at discrete cellular locations (Table S3). We used Fisher's exact test with the Benjamini-Hochberg multiple hypothesis correction [102] to evaluate the significance of the observed differences in localization patterns between pairs of RNAs. The results are schematized in Figure 3B. For example, in the first ideogram of Figure 3B, the cumulative localization of *BBC1:MS2* at cellular structures across all cell cycle stages is compared to the corresponding results for the *ARG3:MS2* localization profile (comparisons for individual stages of the cell cycle are available in Table S3 and Figure S1). Discrete mRNA localization patterns in each comparison were color-coded to reflect significant relative over- or under-representation, with differences at an FDR of 1×10^−2^ represented by light red or blue, respectively, ranging to differences at an FDR of 1×10^−16^, represented by dark red or blue, respectively. When an mRNA's observed localization did not fall into any of the specific categories (presumptive bud, bud, bud neck, cell cortex, actin patch, nucleus, or vacuole), it was assigned the general designation of “cytoplasm”. It should be noted that this default category could encompass a diverse set of specific cellular structures that are not discernible in these images.

Compared to the control mRNA, *ARG3*, *ASH1* and AP mRNAs were enriched at the actin patch; *BBC1*, *LAS17*, and *ASH1*, but not *VRP1*, were also enriched at the cell cortex (the first four ideograms of Figure 3B). The apparent enrichment of *VRP1* at the nucleus could reflect localization of nascent transcripts at the nuclear periphery [102], mRNAs in transit or docked at the nuclear pore complex, or mRNAs in association with perinuclear endoplasmic reticulum (ER), which is semi-continuous with the nuclear envelope.

In addition, some differences in localization were seen among the AP mRNAs. *BBC1* mRNAs, for example, were enriched relative to *ARG3* mRNAs at the presumptive bud (Figure 3B, first ideogram), a site where actin patches cluster during bud formation in the early S phase of the cell cycle [103], [104], [105]. In contrast, *LAS17* mRNAs, but not *BBC1* or *VRP1* mRNAs, were enriched in the definitive bud. *BBC1* mRNA localization to the actin patch and cell cortex was also more pronounced than that of *VRP1* mRNA (Figure 3B, ideogram 9), while both *LAS17* mRNAs and *VRP1* mRNAs were more frequently localized to the nuclear compartment than *BBC1* mRNAs. *ASH1* mRNAs were preferentially localized to the bud and bud neck, consistent with earlier reports [6], [7], [8], when compared to either *ARG3* (Figure 3B, ideogram 4) or the AP mRNAs (Figure 3B, ideograms 5–7). Thus, while *ASH1* mRNA and the AP mRNAs localized to actin patches in a similar fraction of cells (Figure 3A), the specific sites in the cell where this interaction took place were quite different.

Addition of latrunculin B dramatically decreased the frequency of *BBC1* mRNA localization to the presumptive bud and actin patch (Figure 3B, ideogram 11), but not the overall frequency with which it localized to the cell cortex. More tellingly, deletion of *MYO3* results in a redistribution of *BBC1* and *LAS17* mRNAs from the presumptive bud, bud, cell cortex, and actin patches to the cytoplasm, indicating that Myo3 is critical for association of these AP mRNAs with actin patches and at particular sites within the cell. Surprisingly, deletion of *MYO3* also resulted in a shift in *ARG3* mRNA localization from the vacuole to the nucleus, the basis of which is a mystery.

**Figure 4:**
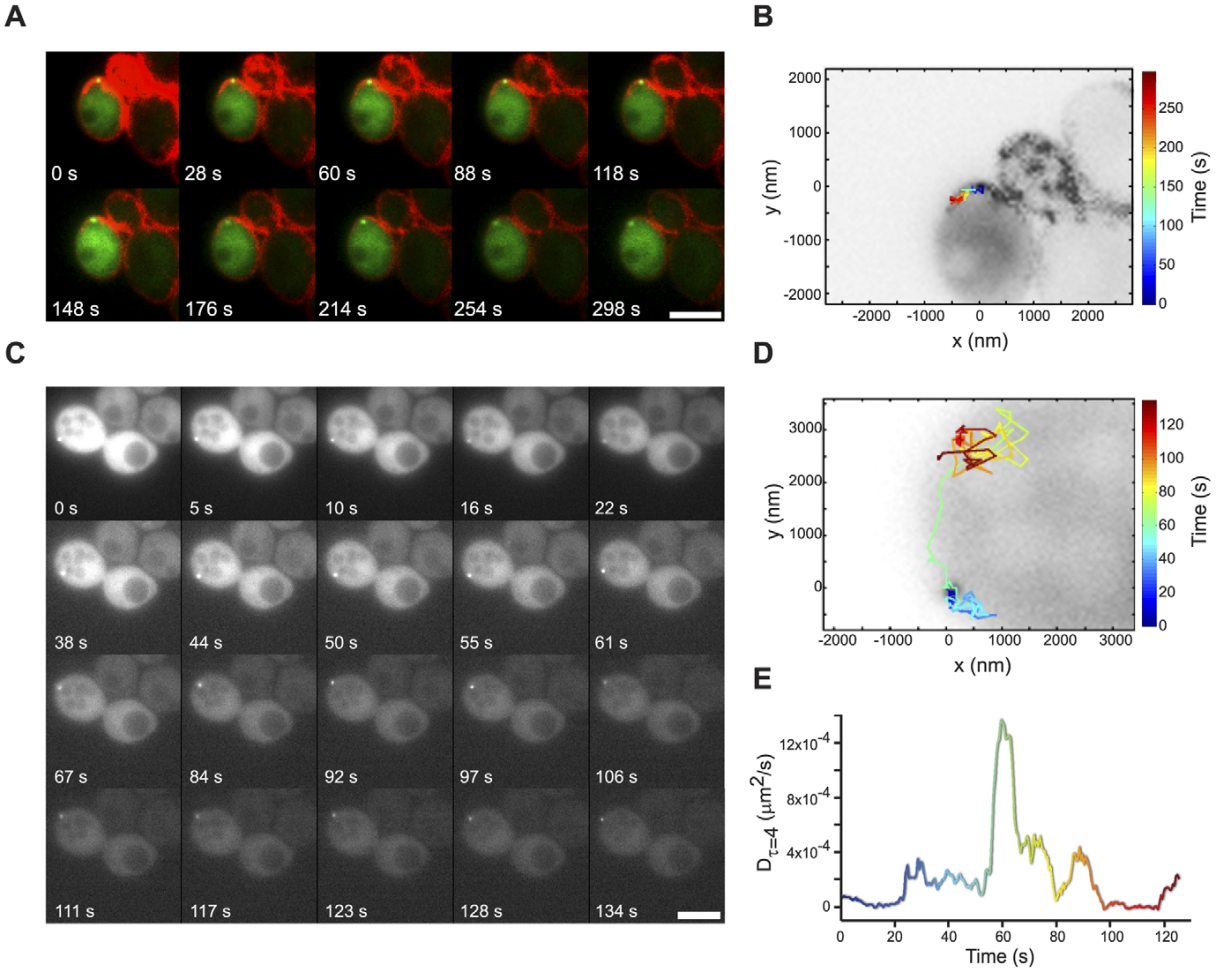
2D dynamics of actin patch mRNA localization. (A) Montage of a time-lapse video of *LAS17:MS2* (green) localization in a haploid, early S phase cell with actin patch accumulation at the presumptive bud indicated by FM4–64 staining (red). Scale bar is 2 µm. (B) The trajectory of the mRNA particle in panel (A) is shown on a grayscale image of the first timepoint (0 s) with black-white inversion. Time is encoded by color in the heatmap. (C) Montage of a time-lapse video of *LAS17:MS2*. Scale bar is 5 µm. Brightness of the latter timepoints in the montage was adjusted relative to the earliest timepoints for display purposes in order to compensate for photobleaching. (D) The trajectory of the mRNA particle from panel (C) is displayed as in panel (B). (E) Change in the diffusion coefficient of the mRNA particle in panels (C) and (D) over time. The diffusion coefficient for short time lags (τ = 4) is graphed with time encoded by color as in panel (D). D was extracted from 4 non-overlapping segments of the trajectory of length 8.992 s, or 16 timepoints. The highest estimated D value, 0.00138 µm^2^/s, leads to an approximate standard deviation of 5.63×10^−3^ µm^2^/s [135], [136].

To investigate the mechanism of recruitment to actin patches, which are dynamic cortical structures [92], [94], [97], we visualized the *LAS17:MS2* mRNP at the presumptive bud by two-color timelapse microscopy using FM4–64 to visualize sites of endocytosis (coincident with actin patches [106]). In an observation over more than four minutes (Figures 4A and 4B, Video S1), a *LAS17:MS2* mRNP seen at the cluster of actively-endocytosing actin patches at the presumptive bud moved less than 500 nm during the timelapse movie (Figure 4B), strongly suggesting that it was confined or effectively anchored at the actin patch. Indeed, we observed many mRNAs residing at the presumptive bud site for extended periods lasting up to 10 minutes (data not shown).

We frequently observed cortical anchoring interspersed with rapid, erratic movement of the mRNA particle consistent with diffusion, as demonstrated by the *LAS17:MS2* mRNP in the M phase cell in Figures 4C and 4D (see also Video S2). Examination of the particle trajectory (Figure 4D) shown in the montage in 4C reveals that the mRNP can move along the cell cortex over a distance of more than 2 µm between sites of prolonged residency. The apparent short-term, 2D diffusion coefficient (Figure 4E) of this mRNP did not change between 10 to 20 s; it then appeared to be briefly confined from 20 to 55 s, rapidly diffusive between approximately 55 to 70 s, then “recaptured” and relatively immobile from 100 to 120 s, suggesting the possibility of a diffusion and capture mechanism for localization.

### Tracking Single *BBC1* mRNPs with High Spatial Precision in Three Dimensions

**Figure 5:**
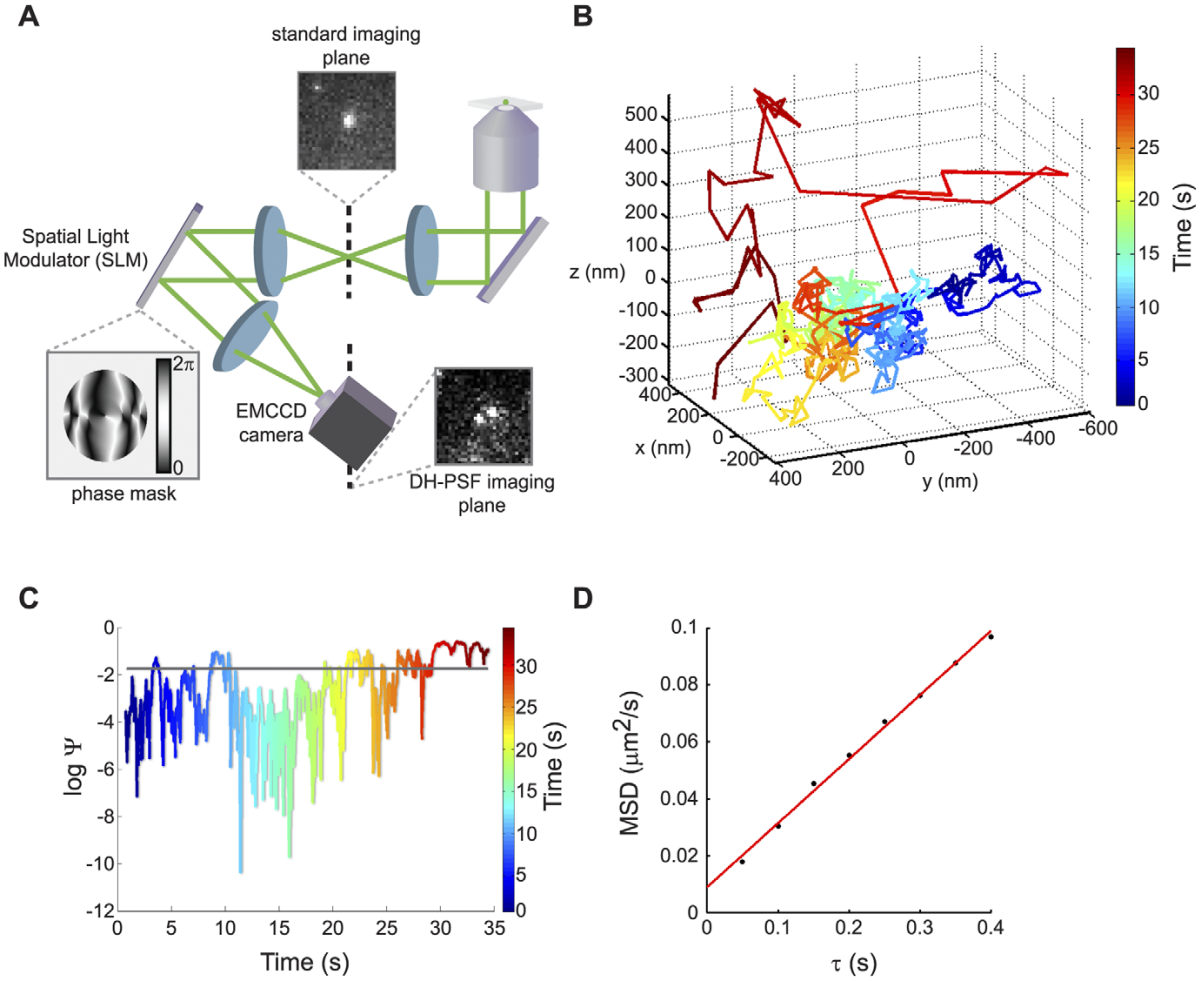
3D super-localization of mRNA particle dynamics using a DH-PSF setup. (A) Schematic of the DH-PSF setup using a spatial light modulator (SLM) exhibiting a phase mask [110] that produces the double-helix point spread function in the imaging plane (EMCCD camera). A single, diffraction-limited point of light shown in the standard imaging plane is detected as two spots in the DH-PSF imaging plane with *z*-position encoded in the angle between the two lobes. (B) 3D super-localization trajectory of a single *BBC1:MS2* particle at 50 ms temporal resolution. Time is encoded by color as displayed in the heatmap. (C) Plot of log Ψ, a measure of the probability that a particle is confined during a portion of the trajectory, for the particle trajectory from panel (B) with time encoded by color. Portions of the trajectory falling below the 95% confidence threshold (dark grey line) are confined. (D) Mean-squared displacement (MSD) plot of the portion of the trajectory in B and C from 29.25 s to 34.45 s, plotted versus time for short time lags.

Preliminary studies (Figure 4) revealing diverse dynamic behaviors of mRNPs at different stages of the cell cycle and within single particle trajectories convinced us that endpoint analyses of localization and analyses of the 2D trajectories with the spatial precision of a standard epifluorescence microscope would provide an inadequate picture of the 3D dynamics of mRNPs. Indeed, standard widefield fluorescence microscopy has localization precision in the axial dimension (500–800 nm) that is several times larger than the localization precision needed to accurately measure fine-scale dynamics, such as localized confinement. For example, our previous work with the DH-PSF system showed that mRNPs exhibiting movement as large as 1.5 µm in *z* could be erroneously described as stationary or confined based on standard 2D widefield fluorescence microscope imaging [107]. To overcome these limitations, we used a double-helix point spread function (DH-PSF) microscope to characterize mRNP dynamics at high temporal and spatial resolution in 3D; the system and method are more thoroughly described elsewhere [107], [108]. Briefly, the DH-PSF microscope uses a spatial light modulator with a phase mask and Fourier plane processing [109], [110] to convert a single, diffraction-limited fluorescent spot, in our case a *BBC1* mRNP, into two spots that are detected in the DH-PSF imaging plane by the electron multiplying charge coupled device (EMCCD) camera (Figure 5A). Translation of the single particle along the *z*-axis is reflected by rotation of the two spots about their midpoint, effectively carving out a double helix within a 2 µm depth of field, thus allowing one to infer the position of a single particle in *z* with high spatial precision based upon the angle between the two spots; *x* and *y* are extracted from the midpoint between the two spots. In practice, the DH-PSF system and image processing allowed us to determine the location of single resolved, diffraction-limited *BBC1* mRNPs to an average spatial precision of 25 nm in *x* and *y* and 50 nm in *z* with 50 ms exposure times, allowing for high confidence studies of single mRNP dynamics.

We chose to focus our further investigation of the dynamics of Myo3-associated AP mRNAs on *BBC1* because of its distinct pattern of localization to the actin patch (Figures 3A and 3B) and good signal to noise for the *BBC1:MS2* mRNP relative to the background MS2-CP-3xEGFP signal in the cytoplasm. A representative, single-particle trajectory of *BBC1:MS2* lasting approximately 35 seconds is displayed in Figure 5B. The mRNP exhibits considerable movement within a relatively confined area, moving a total distance of 28.9 µm within a radius of ∼300 nm from 0–29 seconds, after which the particle exhibits diffusive behavior, moving a total distance of 10.8 µm in just 5 seconds. As discussed below, these prolonged confinements are likely due to an association with actin patches; these events are distinct from the transient (∼1 s) confinements observed in this system and in our previous studies of *ARG3* [107].

A statistical test of the probability that this particle was confined (Figure 5C; [107]) provided evidence for confinement for the majority of the observation period with much of the trajectory surpassing the 95% confidence threshold (Figure 5C, gray line), that is, the probability that the motion is Brownian, Ψ, is very small. The portion of the trajectory from 29.25 s to 34.45 s appeared to be consistent with simple diffusion; analysis of this period by calculating the mean squared displacement (MSD) of the particle over time for short time lags (Figure 5D) resulted in a linear fit of the MSD plot (*R^2^* = 0.996), consistent with the *BBC1* mRNP being diffusive during this period with an average D = 0.038 µm^2^/s.

### Deletion of *MYO3* Decreases the Proportion of Stationary *BBC1* mRNP Trajectories

Although actin patches are dynamic objects [92], [94], [97], the temporal resolution of our measurements (50 ms) make it possible that short term capture in a Myo3- or actin –dependent manner may be detected. Because disruption of *MYO3* and addition of latrunculin B decreased the proportion of AP mRNAs localized to actin patches (Figure 3), we wanted to evaluate the influence of Myo3 and F-actin on the ability of mRNPs to be captured and held “stationary” in cells using statistical tests that we have derived previously for the identification of stationary objects ([107]; Table 1). A trajectory was defined as being stationary if the root mean squared (RMS) displacement at the fourth time lag (0.2 s) was less than the total localization precision of the measurement (62 nm). Because of drift in the microscope, either resulting from the sample or the microscope itself, it is not possible on the current setup to obtain high spatial precision (<50 nm) with an observation time of more than approximately 2–3 minutes. Based on the low-resolution two-dimensional work described above, it was clear that mRNPs bind to actin patches for periods exceeding four minutes; if these events are randomly observed in the DH-PSF studies window, most will be classified as stationary trajectories.

**Table 1:** Differential Proportion of *ARG3* and *BBC1* mRNP Dynamic States.

Type of Motion	*ARG3* *	*BBC1*	*BBC1*+latB	*BBC1 myo3Δ*
Directed	4%	2.2%	2.0%	3.6%
Stationary and Confined	21%	34.4%	27.3%	18.2%
Diffusive	75%	63.4%	70.7%	78.2%

*ARG3 data was published in Thompson et al. [107].

In untreated *BBC1:MS2* cells, 24 of 122 trajectories were stationary. In *BBC1:MS2 myo3*Δ samples, only 10 of 117 trajectories were stationary (*P* = 0.016), indicating that *MYO3* is important for *BBC1* mRNP anchoring. In latrunculin B treated cells, 12 of 104 trajectories were stationary (*P* = 0.10). The difference in stationary events between *myo3Δ* and the latrunculin B treated cells was not significant (*P* = 0.505), suggesting that both perturbations similarly decrease the number of *BBC1* mRNP stationary events. By combining the number of stationary events under both perturbations (22 of 221 trajectories), as both actin and Myo3 may be independent contributors to anchoring, and comparing to the untreated *BBC1:MS2* cells, we conclude that the Myo3-actin interaction is important for *BBC1* mRNP anchoring (*P* = 0.013) as the *BBC1* mRNP is far less likely to be stationary in cells lacking *MYO3* or treated with latrunculin B. *BBC1* mRNPs were more frequently diffusive in latrunculin B treated cells (70.7% versus 63.4%; Table 1) and when *MYO3* was deleted (78.2% versus 63.4%; Table 1). Indeed, in *myo3Δ* cells the distribution of dynamic behaviors observed (stationary, diffusive, and confined) approached that of the control, *ARG3*, suggesting that some *BBC1*-specific confinement behavior is *MYO3* dependent.

### *BBC1* mRNPs Do Not Exhibit Directed, Processive Motion

Although the dynamics of Myo3 motors in *S. cerevisiae* are still poorly understood, the association of a myosin motor protein with an mRNA might suggest directed or active transport of the mRNA molecules. In our previous work, a modified three-dimensional version of the speed correlation index (SCI) was used to identify directed motion [107], [111]. Surprisingly, *ARG3*, which was not found to significantly associate with any motor proteins, displayed 40 events with long periods (>1 s) of actin-dependent directed motion in 267 trajectories [107]. When the SCI tests are applied to *BBC1* trajectories, the long directed movements observed for *ARG3* are absent (data not shown). The average “directed” trajectory for a *BBC1* particle lasted less than 0.7 s, which is within the duration of short Brownian trajectories that would be erroneously described as “directed” by chance. Although Myo3 dynamics are not well characterized, type I myosins like Myo3 are generally thought to be non-processive motors (reviewed in [112]), making Myo3-dependent directed and processive movement over long time periods unlikely. Thus, we suspect that Myo3 may guide *BBC1* mRNAs towards actin patches by non-processive steps that bias the diffusion of the mRNPs towards patches (*vide infra*).

### Deletion of *MYO3* or Addition of Latrunculin B Do Not Affect Apparent *BBC1* mRNP Diffusion Coefficients

**Figure 6:**
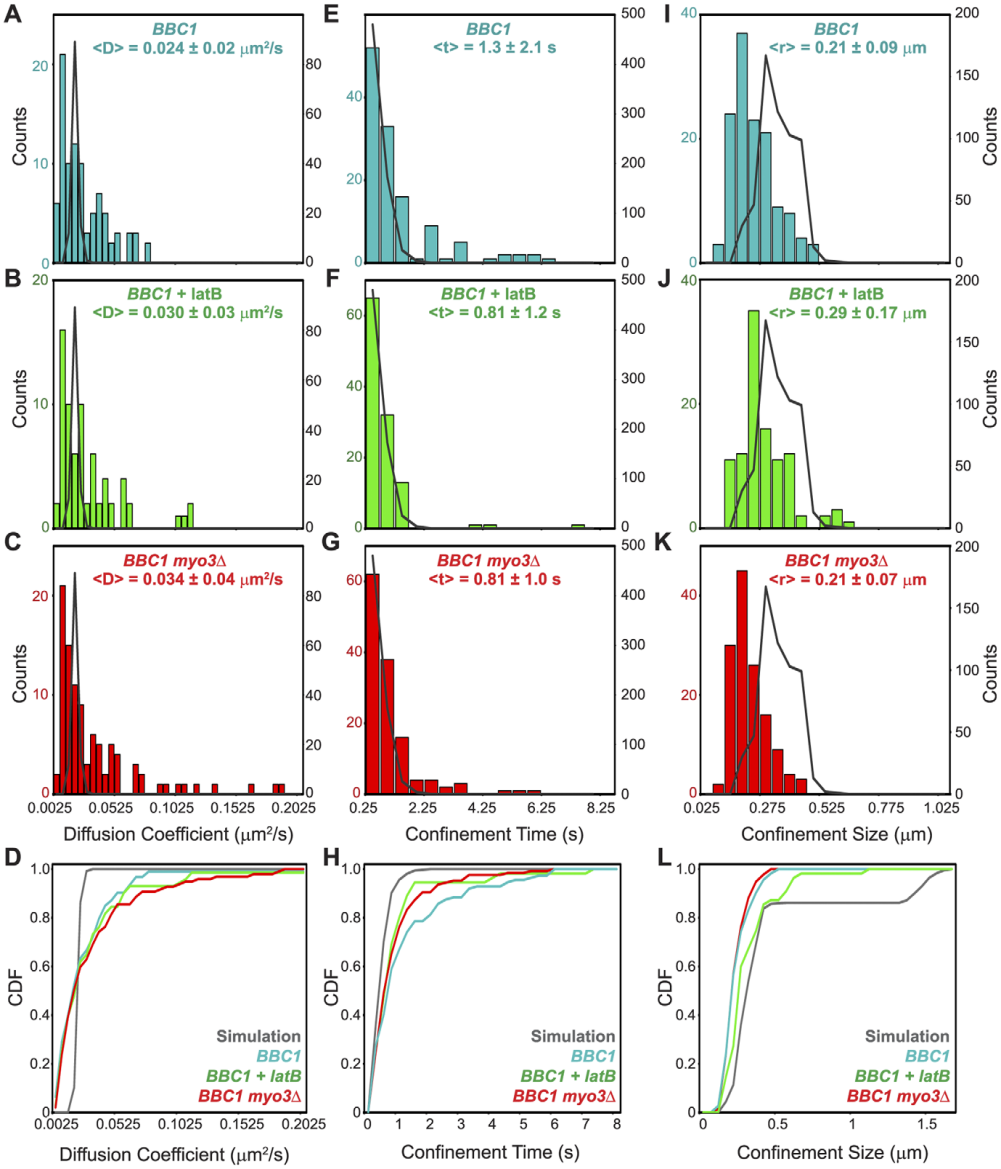
Distributions of diffusion coefficients, confinement times and confinement sizes for *BBC1:MS2* with perturbations. (A–L) Distribution of diffusion coefficients (A–D), confinement times (E–H), and confinement sizes (I–L) for *BBC1:MS2* trajectories in: untreated cells (A, E, I; blue); cells treated with latrunculin B (+latB; B, F, J; green); cells null for *MYO3* (*myo3Δ*; C, G, K; red); and simulations of the values (dark grey curves). Simulations are graphed using the right axes in (A–C), (E–G), and (I–L). (D,H,L) Cumulative distribution function (CDF) plots for diffusion coefficients (data in (A–C)), confinement times ((H); data in (E–G)), and confinement sizes ((L); data in (I–K)). CDF curves are colored as in (A–C), (E–G), and (I–L).

Analysis of the distribution of diffusion coefficients allows us to further quantitatively test the specific impact of Myo3 and F-actin on *BBC1* mRNP dynamics. The average diffusion coefficient for *BBC1* mRNA in latrunculin B treated or *MYO3* null cells is similar to that in unperturbed wildtype cells (D = 0.030±0.03 µm^2^/s, 0.034±0.04 µm^2^/s, and 0.024±0.02 µm^2^/s, respectively; Figure 6A–C and Dataset S3); the distributions were not significantly different by the Kolmogorov-Smirnov (K-S) test or the Mann-Whitney (M-W) test (Table S4). We observed *BBC1* mRNPs with higher apparent diffusion coefficients in *myo3Δ* cells versus wildtype cells (Figure 6C vs. 6A); however, more observations will be required to establish the potential significance of this difference. The apparent diffusion rates of *BBC1* mRNPs were consistently significantly slower that those of *ARG3* mRNPs (0.039 µm^2^/s, *P* = 0.022; [107]); thus, different mRNPs have distinct and characteristic diffusion rates which we hypothesize to reflect differences in mRNP composition and subcellular microenvironment. The distribution of observed apparent diffusion coefficients was much broader than predicted from a model in which the particles and microenvironment are homogeneous (solid curve in Figure 6A–C and labeled “Simulation”; Figure 6D).

### *BBC1* mRNP Confinement on Short Time Scales is Non-Brownian is Affected by Addition of Latrunculin B

As shown in Figure 5B, *BBC1* mRNP exhibits confinement events interspersed with intervals consistent with diffusion, similar to our previous observations of *ARG3* dynamics [107]. In order to determine whether Myo3 or F-actin contributed to the local confinement of *BBC1* mRNPs in live cells, we used equation 1 [91], [107], [113] to quantitatively assess the confinement dynamics of all measured trajectories. 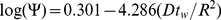
(1)


To analyze the trajectories for confinement using equation 1, the trajectories are first split into sub-trajectories, each with a length, *t_w_*, of 30 time steps (0.45 s). The probability that the observed sub-trajectory reflects *bona fide* confinement or is explained by Brownian motion (i.e. false positive) is calculated as the log of*Ψ*. An MSD analysis is performed on the entire trajectory to obtain the apparent diffusion coefficient, *D*. By then measuring the maximum three-dimensional displacement in each sub-trajectory, *R*, we can now substitute the above values for *t_w_*, *D*, and *R* into equation 1 to obtain the probability, log(Ψ). For each trajectory, 1000 Brownian trajectories with the same length, localization precision, and diffusion coefficient were simulated to calculate a 95% confidence threshold (See Materials and Methods for a more detailed description). When the observed log(Ψ) surpasses (falls below) the threshold, then the subtrajectory is classified as confined, and when log(Ψ) is larger than the threshold then the subtrajectory cannot be significantly distinguished from Brownian motion. In Figure 6, these distributions are denoted as “Simulation”. Results at the 95% confidence threshold are reported here and the test was also performed using 90% and 98% confidence intervals [Dataset S3].

The observed confinement times at the 95% confidence threshold were similar for all three experimental cases: 1.3±2.1 s, 0.81±1.0 s, and 0.81±1.2 s for *BBC1:MS2* cells (untreated), cells null for *MYO3* (*BBC1 myo3Δ*), and cells treated with latrunculin B (*BBC1*++latB; Figure 6E–G and Dataset 3), respectively. Deletion of *MYO3* or addition of latrunculin B did not affect the confinement time (*P* = 0.26 and *P* = 0.19, respectively; Table S5). However, *BBC1* confinement periods in untreated, *BBC1 myo3Δ*, and *BBC1*+latB cells were much longer, on average, than the simulation (0.42±0.3 s; *P*<0.0001, *P*<0.0001, and *P* = 0.004, respectively; Table S5). The significantly shorter confinement times in the simulation are due to short-lived Brownian fluctuations that represent false-positive classification as “confined,” which is similar to previous findings [113]. The significant difference between the observed apparent *BBC1* confinement and simulations indicates that the *BBC1* confinement events are not false positives and cannot be solely described by Brownian dynamics. It should be noted that these non-Brownian confinements were also observed in *ARG3* [107], and might be explained by interactions with structures in the cellular mRNP microenvironment.

For the confinement size distributions, the mean confinement size in each case was 0.21±0.09 µm, 0.21±0.07 µm, and 0.29±0.17 µm for *BBC1* untreated, *BBC1 myo3Δ*, and *BBC1*+latB, respectively (Figure 6I–K, Dataset S3). The confinement size distribution predicted for a particle diffusing in a homogeneous medium with the same apparent diffusion coefficient (D) as *BBC1* (false positives from the Brownian mimic) were considerably larger and more varied with a mean value of 0.45±0.4 µm and the distributions for the three experimental cases were all significantly different from the simulated distribution (*BBC1*, *P*<10^−11^; *BBC 1 myo3Δ*, *P*<10^−12^; *BBC1*+latB, *P*≈0; Table S6). This indicates that *BBC1* binding events restrict the particle to a smaller volume than expected for Brownian fluctuations. The deletion of *MYO3* did not have a significant effect on the observed confinement size (*P* = 0.87), although the addition of latrunculin B did result in significantly larger confinement size when compared to both *BBC1* untreated and *BBC1 myo3Δ* (*P* = 0.001 for both). These results suggest that the effects of Myo3 on *BBC1* mRNP local confinement size are minimal and that when *BBC1* is not confined in an actin dependent manner (e.g. bound to an actin patch), then it is diffusing with intermittent short time interactions, a phenomenon also observed in *ARG3* [107]. However, introduction of latrunculin B increases the volume in which the *BBC1* mRNP is confined, suggesting that actin cables or patches play a significant role in restricting the local dynamics of the mRNPs.

## Discussion

We used a systematic approach to identify the mRNAs associated with the cytoskeletal motor proteins of *S. cerevisiae*. We found consistent association of distinct functionally related sets of mRNAs with 4 of the 5 known yeast myosins, Myo1, Myo2, Myo3, and Myo4, and 2 of the kinesin-like proteins, Kip3 and Kar3. In total, approximately 1,000 mRNAs are associated with cytoskeletal motor proteins in *S. cerevisiae*, demonstrating that mRNA association with motors is pervasive and may be an important means of generating widespread mRNA asymmetries. We also sought to determine the mechanism by which motor-dependent mRNA localization might be occurring. We chose to focus further investigation on the interaction of mRNAs encoding key regulators of actin patch dynamics with a type I myosin, Myo3. We tagged the endogenous *LAS17*, *VRP1*, and *BBC1* loci with 12 MS2 hairpin aptamers which, when expressed in conjunction with the EGFP-tagged MS2 hairpin binding protein, allowed us to visualize mRNP localization in live cells. Using an mCherry-tagged Sac6 protein to mark actin patches, we confirmed that these AP mRNAs localized to actin patches significantly more often than did a control mRNA, *ARG3*, and at a frequency comparable to *ASH1* (Figure 2 and Figure 3). The AP mRNAs associated with actin patches at sites distinct from *ASH1*, with *ASH1* exhibiting a stronger preference for localization to actin patches in the bud or bud neck (Figure 3). The differential localization of AP and *ASH1* mRNAs to actin patches in distinct parts of the cell suggests both that these mRNAs use different mechanisms for delivery or anchoring to actin patches and that the actin patches at different cellular locations may be molecularly distinct. Both Myo3 and F-actin were critical for maintaining AP mRNA localization at actin patches, suggesting that Myo3 is important for either delivery of the AP mRNA to actin patches or anchoring of the transcript at these sites, or both.

### Dynamics of a Myo3-Associated Actin Patch mRNA, *BBC1*

To complement our study of the static localization of mRNAs in live yeast cells and to further investigate the mechanism of AP mRNA localization to actin patches, we combined low resolution, 2D imaging of mRNP dynamics on long time scales with high temporal resolution, 3D super-localization (DH-PSF) microscopy of single particle dynamics on short time scales. 2D imaging results suggested that AP mRNAs associate with sites of actin patch enrichment for extended periods of time (Figure 4), alternating with periods of diffusion, consistent with a diffusion and capture model for localization of AP mRNAs to the actin patch. Because critical features of the dynamic behavior of mRNAs occur in a spatial and temporal scale much finer than can be resolved by conventional epifluorescence microscopy, we used a 3D super-localization microscopy method [107], [108], [114] that provides a spatial precision of 25 nm in *x* and *y* and 50 nm in *z* at a temporal resolution of 50 ms to observe several hundred *BBC1* mRNA trajectories. Neither depolymerization of actin nor deletion of *MYO3* affected the average 3D diffusion coefficient of *BBC1* particles, suggesting that diffusive movement of the particle is not greatly influenced by either of these factors. The distribution of *BBC1* diffusion coefficients was significantly different from that of the control mRNA, *ARG3* (<D> = 0.039±0.02 µm^2^/s, *P* = 0.022; [107]), demonstrating that distinct mRNPs have characteristic dynamic properties, perhaps dependent upon mRNP makeup and local cellular environment.

Deletion of *MYO3* had little effect on the apparent confinement time and volume of the *BBC1* particle (Figure 6). The apparent confinement times of *BBC1* mRNAs in either wildtype or *MYO3* null cells were significantly greater than expected under a model of unconstrained diffusion, indicating that the *BBC1* mRNPs episodically interact with cellular structures that restrict or impede their movement. Although the results were not significant by the Kolmogorov-Smirnov or Mann-Whitney tests with the size of the current dataset, examination of the cumulative distribution function (CDF) plot in Figure 6H demonstrates that deletion of *MYO3* and addition of latrunculin B cause a shift to shorter confinement times, approaching the values obtained in the simulation. This result is consistent with decreased AP mRNA localization to actin patches in *MYO3* null cells (Figure 3) and provides further evidence of a potentially important role for Myo3 and F-actin in the confinement of *BBC1* mRNPs.

Depolymerization of F-actin by the addition of latrunculin B decreased the association of AP mRNAs with actin patches (Figure 3). A significant shift in the distribution of confinement sizes (Figure 6 I–L) indicates that actin plays a critical role in limiting the confinement radius of *BBC1*.

### Model for *BBC1* mRNP Recruitment to Actin Patches

There are several models that could be considered for the apparent roles that Myo3 and F-actin play in the localization and dynamics of the *BBC1* mRNP. First, as Myo3 is an actomyosin motor, one *a priori* model would posit that AP mRNAs undergo Myo3-associated directed transport along actin cables to the actin patches in a manner similar to Myo4-mediated *ASH1* transport to the bud tip [6], [9], [115]. Myo4 is a type V, non-processive motor as a monomer, meaning that there is a low probability that a single Myo4 motor will stay associated with an actin filament through many enzymatic cycles. However, Myo4 becomes processive upon self-assembly into a multi-motor oligomer, suggesting that multiple Myo4 molecules bound to the *ASH1* mRNA cargo facilitate its localization to the bud tip [116]. In contrast, Myo3 is a member of the type I family of non-processive actomyosin motors, which are monomeric, have characteristically slow rates of ATP turnover and actin movement, and thus not expected to be capable of extended directed motion along actin filaments (reviewed in [112], [117]). Indeed, we failed to detect significant sustained and directed motion of the *BBC1* mRNP, even on short time scales.

**Figure 7:**
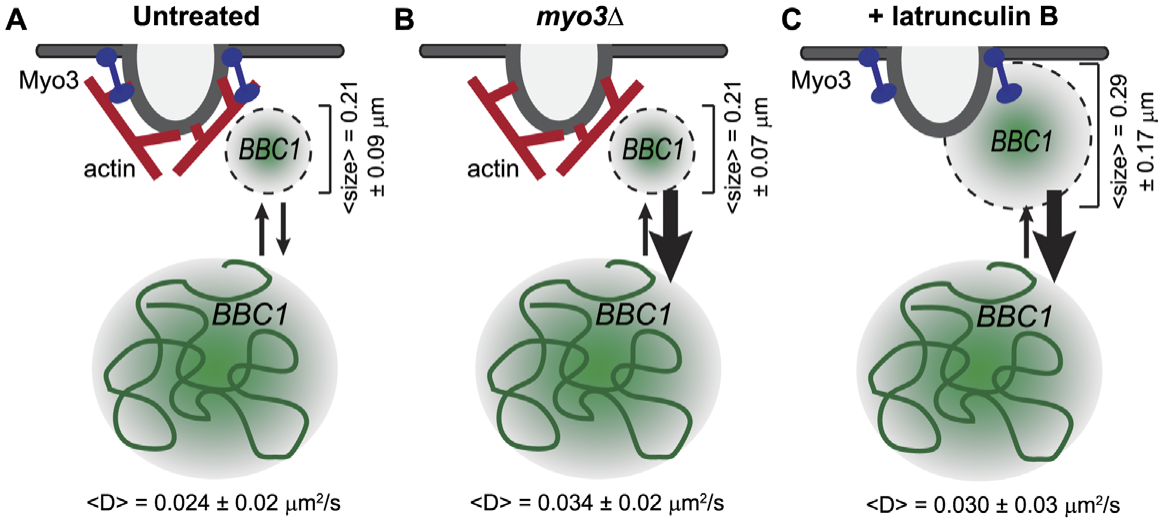
Model of *BBC1* mRNP association with actin patches. (A) *BBC1* can be found in a diffusive state (large green circle) and a confined or stationary state (small green circle) in association with the endocytosing actin patch. Myo3 is represented by a blue rod and actin is colored in red. (B) Deletion of *MYO3* (*myo3Δ*) leads to decreased association of *BBC1* with the actin patch and a decrease in the proportion of *BBC1* mRNPs in the stationary state (large downward arrow). (C) Disruption of F-actin via addition of latrunculin B causes a decrease in the proportion of *BBC1* mRNPs at the actin patch (large downward arrow) and an increase in confinement size (mid-sized green circle). Average diffusion coefficients and confinement sizes are displayed.

A second model for Myo3 and actin-dependent localization involves Myo3 serving solely as an anchor for AP mRNAs at the actin patch. In this model, randomly diffusing AP mRNAs would become trapped, or anchored, at actin patches in an actin- and Myo3-dependent manner, consistent with the decrease in AP mRNA localization to actin patches we observed upon deletion of *MYO3* or treatment with latrunculin B (Figure 3 and 7). Indeed, an anchor-like role for a motor protein has been reported in which a dynein motor was shown to serve as an anchor for proper *gurken* localization in *Drosophila* [30], [31].

A third model posits that Myo3 associated with the *BBC1* mRNP facilitates a biased diffusion towards actin patches. In this model, the saltatory but consistently polarized steps of Myo3 on actin filaments that are themselves polarized toward the actin patches [118], [119] results in a slight but consistent and cumulative bias in mRNP movement towards areas of actin nucleation such as the actin patch. In order to conceptually test this third model, we performed simulations to determine how long a freely diffusing mRNP with the apparent diffusion coefficient of *BBC1* (0.025 µm^2^/s) would take, on average, to reach an arbitrary point on the cell surface representing the cluster of 23 spherical actin patches [81] in an S phase cell, each 140 nm in diameter, while being excluded from the nucleus and vacuole (see Text S1 and Text S2 and example trajectories in Figure S2 and Figure S3). Without a bias in diffusion, we found that the simulated mRNP would require an average of 110 min to reach the actin patch, which is longer than the reported lifetime of the *BBC1* mRNA (*t*_1/2_ = 43 min, [120]) and longer than a full *S. cerevisiae* cell cycle. However, with even a seemingly tiny bias in the diffusion, approximated here by a single 8 nm step for the non-processive motor [121] in the direction of an actin patch once per second, we find that the simulated mRNP would require an average of 11 min to travel from the nucleus to the actin patch. This seemingly negligible bias would not be reflected by an observable change in the overall diffusion coefficient or even, necessarily, in the confinement volume. However, our modeling demonstrates that the diffusion rate of *BBC1* alone is not sufficient to account for the efficient localization of this mRNA to the actin patch by simple, unbiased diffusion. Furthermore, *BBC1* binding to the non-processive motor, Myo3, and the lack of any sustained directed movement are consistent with biased diffusion for efficient localization to actin patches. Indeed, in such a model the increased concentration of polarized actin filaments that arises from the actin nucleation and branching at sites of endocytosis would create a potential well where a particle exhibiting biased diffusion could be effectively trapped without being rigidly bound; sites where actin patches are clustered, such as the presumptive bud in early S phase cells, would trap mRNPs for longer periods of time. Biased diffusion by non-processive motor movement before capture in a region of the cell that is dense with actin filaments is similar to the proposed mechanism of melanosome transport in *Xenopus*. Single particle tracking of melanosomes transported by microtubule motors and by myosin V revealed long, microtubule-based movement interspersed with short, intermittent steps along actin filaments with frequent loss of attachment, passive diffusion, then reattachment to other actin filaments until the melanosomes were tethered in the actin-rich distal region of dendrites [122].

We believe our results favor a biased diffusion model for AP mRNA localization to actin patches. Retention of the AP mRNPs in the potential well of the actin patch would provide time for localized translation of the AP mRNAs to locally enhance actin branching and dynamics. For example, previous work in our laboratory found that in rapidly growing *S. cerevisiae* haploid cells, 69% of *LAS17* (WASP) mRNAs are associated with polyribosomes, averaging 11 ribosomes per mRNA [123]. Assuming a translation rate of 10 amino acids per second [124], a single ∼1900 base *LAS17* transcript in polysomes would yield only about 10 proteins per minute. Because there are only 1–3 *LAS17* transcripts per haploid yeast cell [98], [99], the ability to localize *LAS17* and other AP mRNAs for localized translation at sites of actin branching might confer a significant selective advantage. *β-actin* mRNAs and mRNAs encoding the Arp2/3 components are also localized to sites of actin branching and cytoskeletal rearrangement in mammalian cells [20], [21], [22], [23], [24], [25], suggesting that this may be a common mechanism for localization of mRNAs important for actin polarization.

### Localization and Dynamics as mRNA-Specific Programs

Both the localization pattern and dynamics of *BBC1* and *ARG3* are distinct. The differences could be due to the different interactions with motor systems, local cellular structures, ribosomes, and RNPs. We suspect that differences in mRNP localization and dynamics are encoded in each mRNA by an mRNA-specific arrangement of RBP binding sites, which may also program interactions with the translation apparatus and mRNA decay machinery [5], [43], [46], [120].

Previous studies of *ASH1* localization in yeast have indicated that the actomyosin protein, Myo4, binds the *ASH1* transcript indirectly through the RBP, She2, and the adapter and RBP, She3 [8], [9], [10], [11], [12], [13], [14], as well as the RBPs Khd1 and Puf6, which act jointly to mediate specific transport and to repress translation until the mRNA has reached the appropriate cellular location [41], [42], [125], [126]. Disruption of *SHE2*, *SHE3*, *PUF6*, or *KHD1* impairs proper *ASH1* localization [6], [41], [125], [127]. The precedent set by the *ASH1* system suggests that the specific constellation of RBPs and ribosomes that associate with each mRNA may have distinct and important roles in mRNA localization. The full molecular system that executes the localization program of AP mRNAs remains to be defined, although we suspect that one or more RBPs may act as sequence-specific adapters between Myo3 and the AP mRNAs. The definition of such a molecular system will be a critical step toward understanding how the localization pattern and dynamics of the AP mRNAs are encoded and executed and how they contribute to fitness.

In the model we favor for AP mRNA localization, the non-processive motor, Myo3, promotes asymmetric localization of AP mRNAs using a “parkour-like” mechanism that uses actin filaments it encounters by diffusion as platforms for saltatory, directed movements, to introduce a directional bias to the diffusion of AP mRNPs. We hypothesize that the “parkour” mechanism could be a simple and widespread mechanism by which non-processive motors can localize mRNAs and other cargoes to diverse cellular sites. Indeed, the abundance of non-processive motors in other organisms combined with the diversity of mRNAs that might localize to distinct locations within the crowded and heterogeneous cellular environment might necessitate such a simple and adaptable means of achieving asymmetry without the traffic jams and queueing that could impair the efficiency of processive motors. New analytical tools will need to be developed in order to detect and verify what will likely be subtle biases in path for non-processive, motor-associated mRNAs and other asymmetrically localized cellular components.

## Materials and Methods

### RNA immunoaffinity purifications

#### Formaldehyde crosslinking of motors

250 ml cultures of S. cerevisiae strains containing 9Myc- or GFP-tagged motor proteins were grown to mid-log phase in YPD medium then incubated with 1% formaldehyde (v/v) for 20 min before collection by centrifugation. Cell pellets were washed with 25 mls and then 10 mls of cold Tris-buffered saline (20 mM Tris-HCl [pH 7.5], 150 mM NaCl). Pellets were then frozen in LN_2_ and stored at −80°C. For lysate preparation, pellets were resuspended in 450 µl of lysis buffer (50 mM HEPES-KOH [pH 7.5], 140 mM NaCl, 0.1% Nonidet-P40, 1 mM EDTA, 0.1% sodium deoxycholic acid, 1 mM PMSF, 1 mM DTT, 0.1 U/ul SUPERase-In [Applied Biosystems], and 12.5 µg/ml each of pepstatin, leupeptin, aprotinin, antipain, and chymostatin) and mechanically disrupted with glass beads in a mini-beadbeater-8 (Biospec Products). The insoluble pellet was recovered and suspended in 1.8 ml of lysis buffer then sonicated using a Branson Sonifier with settings at 50% duty cycle and at the microtip maximal power setting for three minutes with rests on ice to prevent heating of the sample. The sonicated extract was cleared by centrifugation at 4,000 g for 1 min and the soluble portion was transferred to a new tube; 50 µl of the extract was removed for reference RNA isolation. 100 µl of pan-mouse IgG Dynal magnetic beads (Invitrogen) was pre-blocked with 5 mg/ml BSA in cold phosphate-buffered saline (PBS) and pre-incubated with either 10 µl of 9E11 monoclonal anti-c-myc antibody (Lab Vision) or JL-8 (anti-GFP) Living Colors A.v. monoclonal antibody (Clontech) overnight. Beads were washed with 5 mg/ml BSA in PBS and pre-equilibrated in lysis buffer before addition to the lysate (∼1.6 ml). Bead/lysate suspensions were incubated for 4 h at 4°C with gentle rotation. Beads were collected with a magnet and washed two times with 1 ml of lysis buffer, then two times with 1 ml of lysis buffer with increased sodium chloride concentration (360 mM NaCl final). Protein/RNA complexes were eluted from the beads by the addition of 100 µl of elution buffer (50 mM Tris-HCl [pH 8.0], 10 mM EDTA, 1% SDS, 0.04 U/µl SUPERase-In, and 10 mM DTT) and incubation at 70°C for 45 min with shaking. 50 µl of the reference extract was combined with 150 µl of elution buffer and also incubated at 70°C for 45 min with shaking. RNA was purified using the RNeasy Mini Kit (Qiagen) with on-column DNAse digestion following manufacturer's instructions.

#### Latrunculin B purification of motors

Immunopurifications of 9Myc- or GFP-tagged motor proteins from cultures treated with latrunculin B were carried out in a manner similar to that described for crosslinking cells with some modifications. 250 ml cultures were grown to mid-log phase, harvested by centrifugation, and washed with 25 mls and then 10 mls of cold Tris-buffered saline. Pellets were placed on ice and resuspended in 600 µl of lysis buffer with the addition of 2 µg/ml latrunculin B, then mechanically disrupted by a beadbeater. Lysates were cleared by centrifugation at 4,000 g for 1 min at 4°C, the volume was brought up to 1.4 ml using lysis buffer, and 200 µl was removed for reference RNA isolation. 100 µl of pan-mouse IgG Dynal magnetic beads pre-incubated with antibody (see above) was combined with the remaining lysate and incubated for 4 h at 4°C with gentle rotation. Washes were performed as described above. Protein/RNA complexes were eluted with elution buffer (see above), incubation at 70°C for 10 min, and vigorous vortexing. Immunopurified and total lysate RNA were extracted with phenol∶chloroform∶isoamyl alcohol (25∶24∶1; Invitrogen) twice and chloroform once using 2.0 ml Phase Lock Heavy tubes (Eppendorf) followed by ethanol precipitation in the presence of 7.5 µg Glycoblue (Ambion) as carrier.

### Microarray sample preparation, hybridization, washing, and scanning

The entire RNA eluate (<1 µg) from immunopurifications and 1 µg of total RNA purified from lysates were amplified using the amino allyl messageamp II RNA amplification kit (Applied Biosystems) following manufacturer's instructions with minor modifications. 20 ng of doping control RNAs (Stanford Functional Genomics Facility) were spiked into the labeling reactions to aid in microarray scanning. The entire product of IP-enriched RNA amplification and up to 8 µg of the amplified reference RNA product were covalently labeled with Cy5 or Cy3 NHS monoesters (GE Healthcare Life Sciences), respectively. The combined dye-labeled probes were diluted in a 50 µl solution containing 2.5× SSC, 20 mM HEPES-KOH (pH 7.3), 30 µg of poly(A) RNA (Sigma), and 0.2% SDS. The sample was incubated at 95°C for 5 min then centrifuged at 14,000 *g* for 5 min before application to microarrays. Custom microarrays were designed and spotted as previously described (Hogan et al., 2010). Further information about the probes used in printing the arrays is available from the Operon Web site (https://www.operon.com/; S. cerevisiae YBOX V1.0) and detailed protocols for microarray experiments can be found on the Brown lab website (http://cmgm.stanford.edu/pbrown/protocols/index.html). Microarray processing prior to hybridization to poly-lysine and epoxysilane glass slides, hybridization using the MAUI hybridization system (BioMicro), as well as post-hybridization washing was performed as previously described [46], [71]. Microarrays were scanned using either an AxonScanner 4200 or 4000B (Molecular Devices) and PMT levels were adjusted to achieve 0.1% pixel saturation. Microarray spots were located and analyzed using Genepix Pro 5.0 or 6.0 software (Molecular Devices) and intensity data was submitted to the Stanford Microarray Database [128] for further analysis.

### Yeast Strain Construction

#### Strains for Immunopurification

The haploid yeast strain PSY2156/JCY18 (MATa ade2-1 trp1-1 can1-100 leu2-3,112 his3-11,15 ura3 GAL+ PSI+; [129]) was transformed with a 9Myc klTRP1 C-terminal tagging cassette described previously [44]. The 9x MYC C-terminal tagging sequence was integrated at the endogenous loci to generate JCY19 (myo4:9Myc), JCY31 (smy1:9Myc), JCY36 (kip2:9Myc), JCY37 (dyn1:9Myc), JCY38 (myo3:9Myc), JCY39 (myo5:9Myc), and JCY304 (myo2:9Myc). Candidates were confirmed by western blot of total cell lysates using a polyclonal rabbit anti-C-myc (A-14) antibody (Santa Cruz). C-terminal GFP-tagged protein strains [45], including JCY33 (myo4:GFP), JCY34 (smy1:GFP), JCY41 (kip3:GFP), JCY42 (myo2:GFP), JCY43 (vrp1:GFP), JCY44 (las17:GFP), JCY45 (myo5:GFP), JCY46 (kar3:GFP), JCY49 (myo1:GFP), JCY50 (myo3:GFP), JCY51 (kip2:GFP), JCY57 (kip1:GFP), JCY59 (dyn1:GFP), and JCY60 (kar9:GFP) were obtained from Invitrogen. Strains were confirmed by western blot of total cell lysates using a polyclonal rabbit anti-GFP antibody (Living Colors Full-Length A.v., Clontech). Strain genotypes are listed in Table S1.

#### Strains for Microscopy

We chose to use an MS2 system in which the endogenous transcript is expressed from the native promoter since previous studies have indicated that BBC1, LAS17, and VRP1 are expressed at just a one to three copies per cell [98], [99], raising concerns about potential spurious results from overexpression of a chimeric transcript from an exogenous vector. In addition, a number of studies have indicated that localization information is frequently found in the 3′-UTR of transcripts [2], [3], necessitating a method that would retain the native 3′-UTR sequence. Strains expressing ARG3, ASH1, BBC1, LAS17, and VRP1 with 12 MS2 hairpins inserted between the open-reading frame and 3′ UTR were constructed as described in Haim-Vilmovsky and Gerst [130]. The loxP-SpHIS5-loxP-MS2L cassette was amplified from pLOXHIS5MS2L using gene-targeting primers (Table S7). The tagging cassette was integrated into the haploid yeast genome of JCY66 (MATa ade2-1 trp1-1 can1-100 leu2-3,112 his3-11,15 ura3) as described in Thompson et al. [107] and candidates were screened via PCR of genomic preparations using appropriate primers (Table S7). Successful candidates were transformed with the pSH47 plasmid [131] followed by overnight galactose-induced expression of the Cre recombinase in liquid media. Candidates were screened for the successful recombination of the loxP sites and removal of the selectable SpHIS5 marker by the inability to grow on SC his^−^ media and by PCR of genomic preparations using appropriate primers (Table S7). The resulting strains were then transformed with the pMS2-CP-GFP(x3) plasmid, allowing methionine-inducible expression of the MS2 coat protein (MS2-CP) fused to three copies of EGFP (MS2-CP-GFPx3) in order to create JCY324 (arg3:MS2), JCY107 (ash1:MS2), JCY233 (bbc1:MS2), JCY178 (las17:MS2), and JCY115 (vrp1:MS2).

The C-terminal Sac6:mCherry tagged strain (*sac6:mCherry*) was constructed by amplifying the mCherry KanMX4 cassette from pBS34, obtained from Dr. Roger Y. Tsien, University of California, San Diego, via the Yeast Resource Center (YRC) at the University of Washington, using primers that include sequences complementary to those flanking the stop codon of *SAC6* (Table S7). The *mCherry:Kan* cassette was then transformed into JCY93, also known as EY0987 ([45]; *MATα his3Δ1 leu2Δ0 lys2Δ0 ura3Δ0*), to create JCY230. Candidates were selected on plates containing 400 µg/ml G418 (Sigma-Aldrich) and confirmed by western blot using a polyclonal rabbit anti-red fluorescent protein antibody (Rockland) and microscopy. The *sac6:mCherry* strain, JCY230, was mated to JCY324 (*arg3:MS2*), JCY107 (*ash1:MS2*), JCY233 (*bbc1:MS2*), JCY178 (*las17:MS2*) and JCY115 (*vrp1:MS2*) to create the diploid strains JCY335, JCY255, JCY251, JCY267, and JCY263, respectively. In order to assay mRNA localization to FM4–64 internalizing actin patches, the haploid *vrp1:MS2* (JCY115) strain was mated to a wildtype *MATα* strain (JCY93) to create JCY126.

In order to construct diploid homozygous null *MYO3* (*myo3Δ*) strains, two different *MYO3* knockout cassettes were used. The *myo3Δ::KanMX4* cassette was PCR amplified from a genomic preparation of the *myo3Δ* strain in the *S. cerevisiae* knockout collection (JCY179; [132]) using primers complementary to sequences several hundred bases upstream and downstream of the disrupted *MYO3* locus (Table S7). The *myo3Δ::KanMX4* cassette was transformed into *las17:MS2* (JCY178), *bbc1:MS2* (JCY233), and *arg3:MS2* (JCY324), selected on plates containing 400 µg/ml G418 (Sigma-Aldrich), and confirmed by PCR of genomic preparations to create strains JCY395, JCY396, and JCY411, respectively. The *myo3Δ::hph* (hygromycin B resistance) cassette was amplified from pBS35 (Dr. Roger Y. Tsien and the YRC) with primers that were designed to include targeting sequences flanking the *MYO3* genomic locus (Table S7). *myo3Δ::hph* candidates of the *sac6:mCherry* strain (JCY230) were selected on plates containing 500 µg/ml hygromycin B (Clontech) and confirmed by PCR of genomic preps to obtain JCY392 (*sac6:mCherry myo3Δ:hph*). JCY395, JCY396, and JCY411 were mated to JCY392 to obtain the diploid homozygous null *MYO3* (*myo3Δ*) strains JCY405, JCY406, and JCY420, respectively.

### Sample Preparation for Microscopy

#### 2D Microscopy

Diploid strains for assaying localization of MS2-GFP labeled mRNAs to Sac6:mCherry-labeled actin patches were grown overnight at 30°C in synthetic complete media lacking histidine (SC his^−^) and supplemented with 40 µg/ml adenine hemisulfate (ade^+^) and 200 µg/ml G418 (G418^+^). Strains were back-diluted into fresh SC his^−^ ade^+^ G418^+^ media and grown until early log-phase, then collected and resuspended in SC his^−^ ade^+^ G418^+^ media lacking methionine (SC his^−^ met^−^ ade^+^ G418^+^) to induce expression of the MS2-CP-GFPx3 protein. Strains were induced for 1 to 3 hrs at 30°C, then collected and resuspended in SC his^−^ ade^+^ G418^+^. Strains were grown for 2 to 3 hrs at 30°C to turn off expression of the MS2-CP-GFPx3 protein in order to decrease cytoplasmic GFP background. Immediately before imaging, 1 ml of culture was washed three times with 1 ml of SC his^−^ ade^+^ G418^+^ and concentrated in a volume of 100 µl. Gelatin pads composed of 25% solid gelatin (Sigma) dissolved in SC his^−^ ade^+^ G418^+^ were made by sandwiching between two pre-cleaned Superfrost microscope slides (Fisher Scientific). After solidification of the gelatin, one slide was removed, 2 µl of culture was applied to the pad to immobilize the cells, and a 22 mm×22 mm #1.5 coverglass (VWR) was applied and sealed with molten valap (1∶1∶1 vaseline, lanolin, and paraffin wax). Fluorescent images were acquired using a 100×1.40 NA PlanApo objective and a CoolSnap-HQ CCD camera (Photometrics) on a Nikon Eclipse 80i microscope.

For experiments utilizing latrunculin B, cells were treated with 500 µM latrunculin B (0.5% DMSO final concentration) immediately before application to gelatin pads. As Sac6:mCherry-labeled actin patches began to recover after 45 min, cells were imaged for less than 30 min before new cells were treated with latrunculin B and imaged; cells treated with 0.5% DMSO alone served as a control.

Diploid strains for assaying localization of MS2-GFP labeled mRNAs to FM4–64-labeled actin patches were grown as described above with the exception that G418 was left out of the growth media. After induction was halted, 100 ul of cells was pelleted and resuspended in SC his^−^ ade^+^ media plus 8 µM FM4–64. The cell suspension was quickly spotted on a gelatin pad, sealed with valap, and timelapse acquisition was started.

### DH-PSF Microscopy

Cells were grown overnight in synthetic complete (SC) his- media supplemented with glucose (2% final wt/vol) and 40 µg/ml adenine hemisulfate. Cells were transferred into induction medium lacking methionine upon entry into early log phase and grown at 30°C for 3 h. Cells were then removed from induction medium for 3 h prior to imaging, washed three times in fresh non-inducing media, and then 2 µl of 10× concentrated cells was spotted onto an agarose pad. Agarose pads composed of 1.5% wt/wt solid agarose (Sigma type 1-A, low electroendoosmosis (EEO)) dissolved in SC his- ade+ were created by sandwiching between two plasma-etched 30 mm×50 mm #1 glass coverslips. After removal of one of the coverslips and deposition of cells, a plasma-etched 25×25 mm glass coverslip was placed on top of the cells to form the imaging interface and then sealed with paraffin wax. Because the stage drift in the microscope during the time scale of our experiments (5–10 s) is negligible compared to the motion of the mRNPs, fiduciary markers were not used.

The DH-PSF 3D microscopy of single mRNP dynamics was performed exactly as previously described ([107] and accompanying Supplemental Methods) except that 50 ms exposure times were used to image *BBC1* mRNP dynamics. To analyze the data, a tracking algorithm based on fitting the observed data to the sum of two Gaussians was employed. The fitting function contained the sum of two Gaussians plus an offset (for background), which allowed for the extraction of an (*x*, *y*) position from the midpoint between the two and the *z*-position from the angle between the two lobes relative to horizontal (see following discussion of DH-PSF calibration). For each frame after the first, the initial conditions used in the fit were taken from the fit in the previous frame. In this way, the particle is automatically tracked by the fitting code. Because only 0–2 mRNAs are found in each cell, it is highly unlikely that there was confusion in the identity of the particle of interest.

Similar to previous work [107], two criteria were then applied to remove spurious position measurements from the trajectories extracted from the fitting algorithm. First, any localization that fell outside the 2 µm depth of field was rejected because the DH-PSF is only designed to accurately measure *z* positions within this region. Second, points were probabilistically rejected by requiring that the calculated velocity between subsequent localizations is less than 4800 nm/s; this removes false localizations because of the high and variable background noise inherent in the MS2 labeling scheme. This velocity was chosen because it is approximately two standard deviations above the mean expected root-mean square velocity from the mean observed diffusion coefficients in these experiments.

### DH-PSF calibration

A calibration of angle versus *z* position was performed by moving 200 nm fluorescent beads (Invitrogen Fluospheres 505/515) immobilized in 1% poly(vinyl) alcohol on a glass coverslip using an objective *z*-positioner (PIFOC *p*-721.CDQ stage with E625.CO analog controller, Physik Instrumente) in 30 steps spaced by 50 nm in *z*. Ten (10) frames were collected at each *z* position. Images of putative beads were identified by hand and cut into 9 by 9 pixel squares followed by manual identification of the two DH-PSF lobes. The data in the box were fit using the Matlab function fminsearch with least squares as the minimization function, exactly as described in Thompson, et al. [114]. We then obtained an angle vs. *z* position curve as well as *x* and *y* calibration curves. Because of aberrations in the system, an *x* and *y* correction is necessary for accurate three-dimensional localization.

Because calibrations are performed using refractive index-matched media, they do not account for the spherical aberration that inevitably occurs in all mRNA imaging experiments in water. In previous work [107], the effect of spherical aberration on the estimation of *z* position was measured for imaging of mRNAs in yeast. As is now well known [133], spherical aberration affects the standard PSF differently above and below the focal plane. Briefly, by measuring the diffusion coefficients in *x*, *y*, and *z* both above and below the apparent focal plane in each trajectory we obtained β factors of 0.88 and 0.66 above and below the focal plane, respectively. Then the true z position, *z*, can be obtained from the apparent *z* position, *z′*, by the following formula 
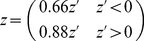
(2)A more detailed description as well as theoretical calculations can be found in the supplemental information of previous work [107].

### Detection and characterization of confinement events

In previous work [107], we built upon work by Saxton, et. al and Simson, et al. [113], [134] and derived an equation for the probability that a purely random (Brownian) diffuser would stay within a given three-dimensional radius given an observed diffusion coefficient and time window. As discussed in the results, the logarithm of the probability, log(Ψ) can be approximated as a linear function given by
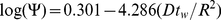
(1)where t_w_ is the length of each subtrajectory, *R* is the maximum displacement in the subtrajectory, and *D* is the measured short time diffusion coefficient averaged over the entire trajectory. The maximum value of *R*, the diffusion coefficient *D*, and the subtrajectory time window, *t_w_*, are used to calculate the probability, Ψ, that a random walk would stay within a circle of that radius. For each trajectory, the average diffusion coefficient and localization precision were measured and Brownian simulations were run to determine the 90%, 95%, and 98% thresholds for confinement. To calculate log(Ψ), the trajectory was segmented into sub-trajectories that were 30 time points long. For each sub-trajectory, a maximum displacement *R* is measured from which log(Ψ) can be calculated in each window. If the calculated log(Ψ) is less than the threshold than the particle is denoted as confined. The confinement radius is the average maximum displacement, *R*, during the confined time window and the confinement time is the time from when the particle first enters a confined state until the particle exits the confined state.

## Supporting Information

Dataset S1 **Significance analysis of microarray results for each immunoaffinity purification approach for the actomyosins, Las17, and Vrp1.**
(TXT)

Dataset S2 **Significance analysis of microarray results for each immunoaffinity purification approach for the kinesin-like proteins and dynein.**
(TXT)

Dataset S3 **Diffusion coefficients and confinement times and sizes for** ***BBC1*** **at different confidence thresholds.**
(XLS)

Figure S1 **Quantification of mRNA localization at cellular loci at all stages of the cell cycle.** Significant differences in localization between mRNAs of interest (columns) at cellular locations (rows) are indicated by color. Cellular locations in cells at different stages of the cell cycle are schematized on the left. Red or blue indicates a –log false discovery rate (FDR) of 2 (light color) to 16 (saturated, dark color), with red colors indicating significant over-representation of mRNA 1 in each pair and blue colors indicating significant over-representation of mRNA 2. Significance was measured by Fisher's exact test.
(TIF)

Figure S2 **Example trajectory of unbiased diffusion of mRNP from model.** An example trajectory for the unbiased diffusion model is displayed along with the outline of a theoretical cell and the total time elapsed before the randomly diffusing mRNP reaches the actin patch. The trajectory is encoded by color with the beginning of the trajectory indicated by red and the end in purple.
(TIF)

Figure S3 **Example Trajectory of Biased Diffusion of mRNP from Model.** An example trajectory for the biased diffusion model, in which the non-processive motor-associated mRNP takes 8 nm steps toward the actin patch once per second, on average, is displayed along with the outline of a theoretical cell and the total time elapsed before the mRNP reaches the actin patch. The trajectory is encoded by color with the beginning of the trajectory indicated by red and the end in purple.
(TIF)

Video S1 ***LAS17:MS2*** **mRNP dynamics in a cell incubated with FM4–64 to reveal endocytic actin patches.** Movie of *LAS17:MS2* mRNP displayed in Figure 4A. Time is displayed in seconds and the scale bar represents 2 µm.
(AVI)

Video S2 ***LAS17:MS2*** **mRNP dynamics.** Movie of *LAS17:MS2* mRNP displayed in Figure 4C. Time is displayed in seconds and the scale bar represents 2 µm.
(AVI)

Table S1 ***S. cerevisiae*** **strains used in this study.** Yeast strains acquired or constructed for this study. Strains JCY18 [129] and GFP strains, including the wildtype parent JCY93 [45], have previously been published.
(XLS)

Table S2 **Percentile rank of mRNAs encoding actin patch associated proteins in IPs of motor proteins.** The average log_2_ Cy5/Cy3 ratio of all mRNAs from IPs of Myo1, Myo2, Myo3, Myo4, Myo5, Las17, and Vrp1 with GFP or 9Myc tags and with the addition of latrunculin B (“latB”) or upon formaldehyde crosslinking (“xlink”) were calculated and ranked. The percentile ranks of all mRNAs from the GO category “actin cortical patch” (GO:0030479) in *S. cerevisiae* are displayed.
(XLS)

Table S3 **Counts of mRNA localization at cellular loci at all stages of the cell cycle.** Counts of mRNA localization for *ARG3:MS2*, *ASH1:MS2*, *BBC1:MS2*, *LAS17:MS2*, and *VRP1:MS2* with and without cellular perturbations at cellular locations during all stages of the cell cycle.
(XLS)

Table S4 ***BBC1*** **diffusion coefficient statistical tests.** Kolmogorov-Smirnov (K-S) and Mann-Whitney (M-W) statistical tests were performed for the distribution of diffusion coefficients (Dataset S3) of *BBC1* untreated, MYO3 null (“*BBC1 myo3Δ*”), and cells treated with latrunculin B (*BBC1*+latB). The two-tailed M-W p-value is reported.
(XLS)

Table S5 ***BBC1*** **confinement time statistical tests.** K-S and M-W statistics at the 90%, 95%, and 98% confidence thresholds are displayed as in Table S3.
(XLS)

Table S6 ***BBC1*** **confinement size statistical tests.** K-S and M-W statistics at the 90%, 95%, and 98% confidence thresholds are displayed as in Table S3.
(XLS)

Table S7 **Oligonucleotide primers used in this study.** Oligonucleotides indicated by an asterisk (*) for generating gene disruptions were published in Winzeler et al. [132].
(XLS)

Text S1 **Description of modeling.**
(DOC)

Text S2 **Code written in R for single particle diffusion modeling in** ***S. cerevisiae*** **.**
(DOC)
