# Supplementary figures and images for: Widespread mRNA Association with Cytoskeletal Motor Proteins and Identification and Dynamics of Myosin-Associated mRNAs in S. cerevisiae
Source: PLoS One. 2012 Feb 16;7(2):e31912. doi: 10.1371/journal.pone.0031912 (PMC3281097; doi:10.1371/journal.pone.0031912)

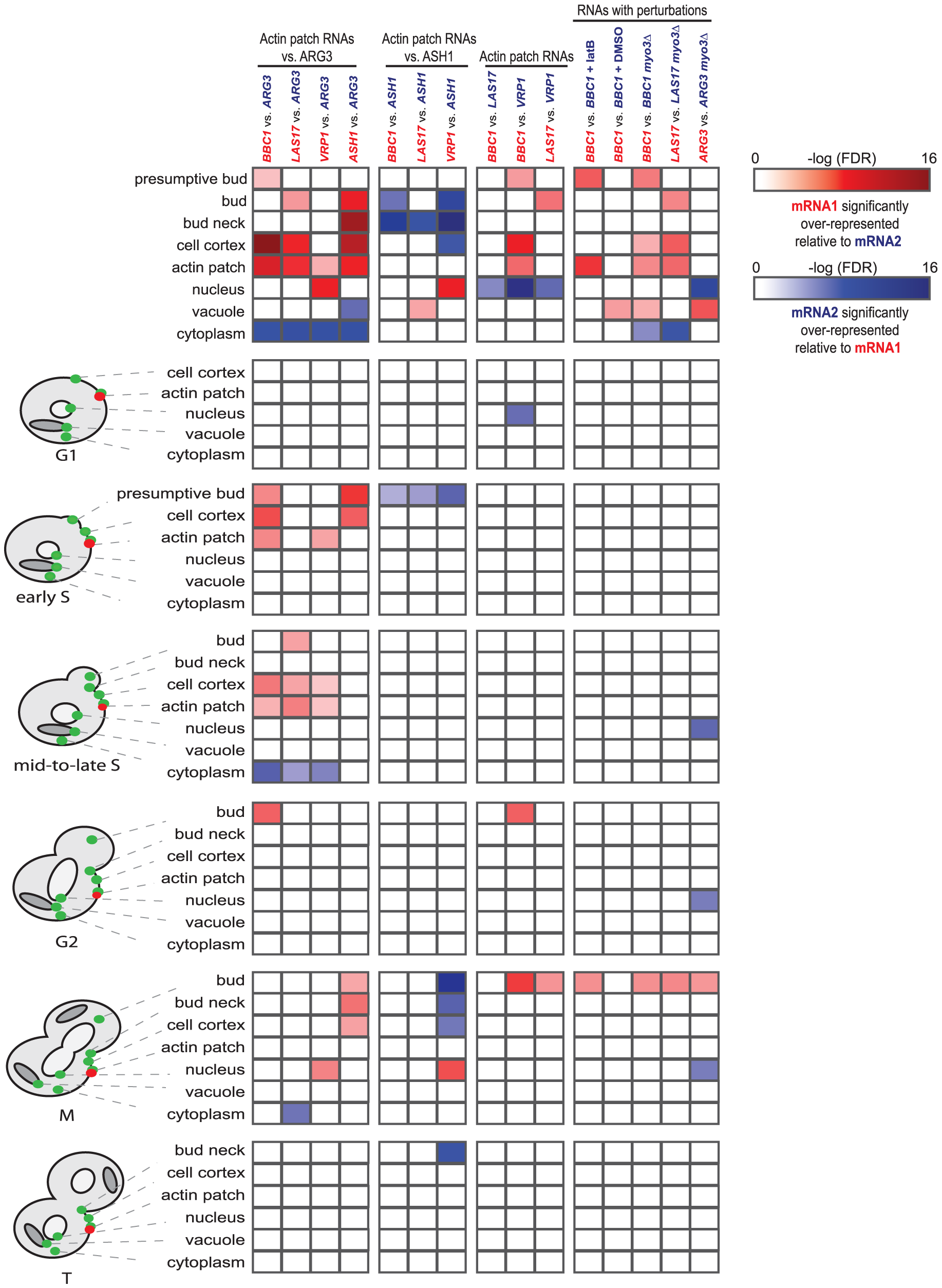

Supplement: Figure S1 — Quantification of mRNA localization at cellular loci at all stages of the cell cycle. Significant differences in localization between mRNAs of interest (columns) at cellular locations (rows) are indicated by color. Cellular locations in cells at different stages of the cell cycle are schematized on the left. Red or blue indicates a –log false discovery rate (FDR) of 2 (light color) to 16 (saturated, dark color), with red colors indicating significant over-representation of mRNA 1 in each pair and blue colors indicating significant over-representation of mRNA 2. Significance was measured by Fisher's exact test. (TIF) [file pone.0031912.s004.tif]

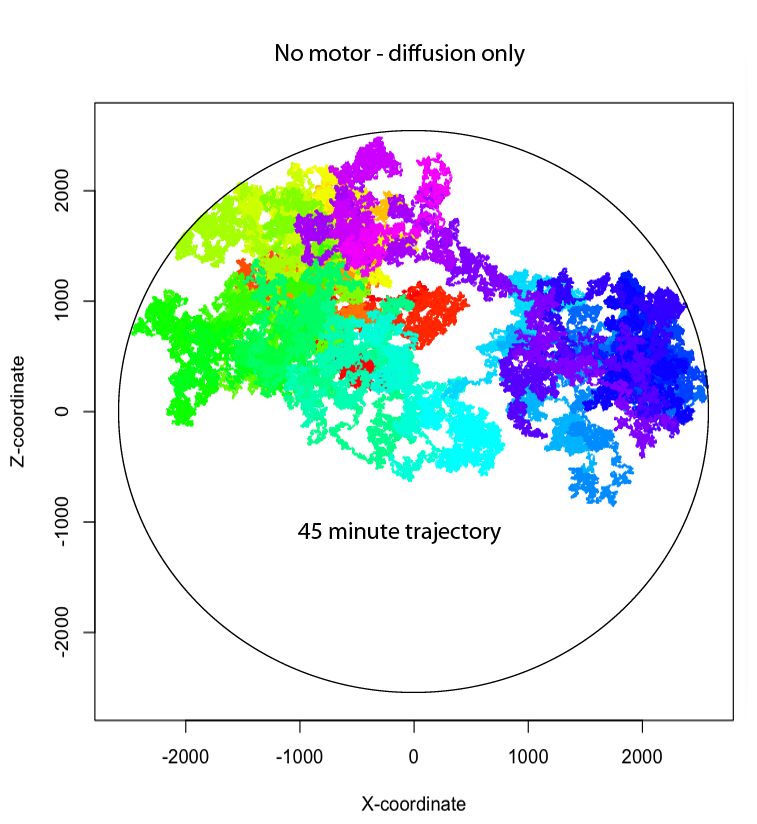

Supplement: Figure S2 — Example trajectory of unbiased diffusion of mRNP from model. An example trajectory for the unbiased diffusion model is displayed along with the outline of a theoretical cell and the total time elapsed before the randomly diffusing mRNP reaches the actin patch. The trajectory is encoded by color with the beginning of the trajectory indicated by red and the end in purple. (TIF) [file pone.0031912.s005.tif]

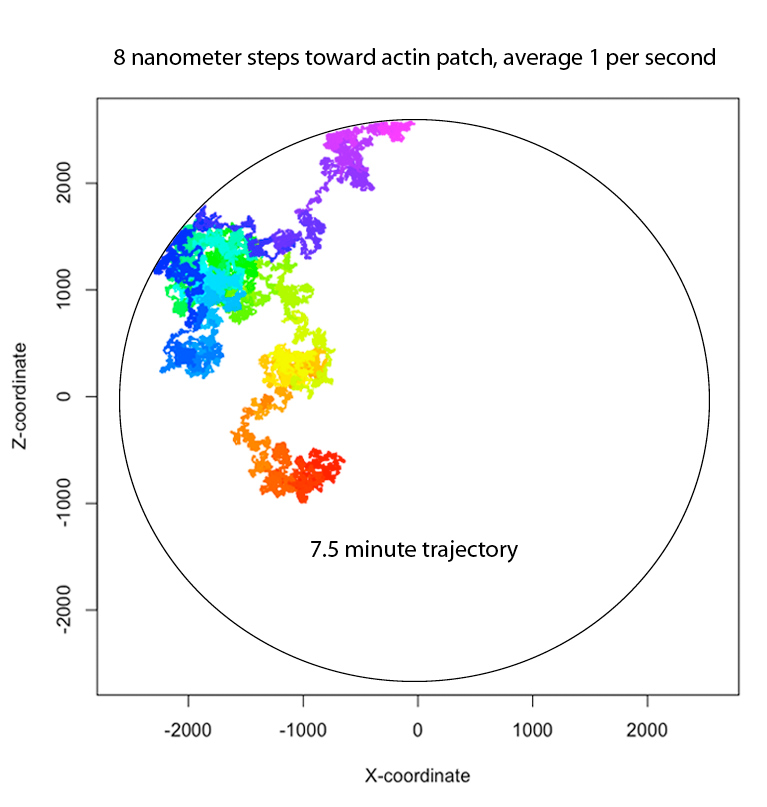

Supplement: Figure S3 — Example Trajectory of Biased Diffusion of mRNP from Model. An example trajectory for the biased diffusion model, in which the non-processive motor-associated mRNP takes 8 nm steps toward the actin patch once per second, on average, is displayed along with the outline of a theoretical cell and the total time elapsed before the mRNP reaches the actin patch. The trajectory is encoded by color with the beginning of the trajectory indicated by red and the end in purple. (TIF) [file pone.0031912.s006.tif]
