# Supplementary material for: Widespread mRNA Association with Cytoskeletal Motor Proteins and Identification and Dynamics of Myosin-Associated mRNAs in S. cerevisiae
Source: PLoS One. 2012 Feb 16;7(2):e31912. doi: 10.1371/journal.pone.0031912 (PMC3281097; doi:10.1371/journal.pone.0031912)
Supplement: Text S1 — Description of modeling. (DOC) [file pone.0031912.s016.doc]

**Description of Modeling**

**Unbiased Model:** In this simulation (see Text S2 for R script) a spherical particle with a diameter of 10 nm, representing the mRNP, diffuses randomly (with D equal to that measured for *BBC1*, 0.025 µm^2^/s) in the homogeneous environment of an S phase cell of 2.6 µm radius (measured from our data). The particle exits the nucleus (0.89 µm radius), located approximately half the distance between the base of the cell and the presumptive bud (both values measured from our data) at a random position and is excluded from the nucleus and the vacuole (0.88 µm radius, measured from our data). The particle diffuses at a root mean squared of 5 nm every millisecond on a random walk and the time it takes for the particle to reach a 400 nm, spherical "actin patch", equivalent in volume to 23 actin patches, each 140 nm in diameter, adjacent to the cell cortex and located at the apex of the cell, as shown in this image, is recorded.

**Biased Model:** The simulation is identical to that for the unbiased diffusion model, except that the freely diffusing particle is transported by a non-processive motor, which takes 8 nanometer steps towards the actin patch, at an average rate of one step per second.
