# Supplementary material for: Widespread mRNA Association with Cytoskeletal Motor Proteins and Identification and Dynamics of Myosin-Associated mRNAs in S. cerevisiae
Source: PLoS One. 2012 Feb 16;7(2):e31912. doi: 10.1371/journal.pone.0031912 (PMC3281097; doi:10.1371/journal.pone.0031912)
Supplement: Text S2 — Code written in R for single particle diffusion modeling in S. cerevisiae . (DOC) [file pone.0031912.s017.doc]

#assign values to the model parameters

numberoftrials<-10

lengthoftrials<-10000000

stepsize<-5#nanometers (specify value for RMS distance per millisecond by diffusion)

motorstep<-8#nanometers (specify value for step length of motor protein)

stepspersecond<-2# (specify value for steps per second by motor protein, in direction of "actin patch")

objectradius<-5#nanometers (radius of the diffusing/transported particle)

targetradius<-70#nanometers (radius of the target - an "actin patch" in this model)

targetnumber<-23 # (number of "actin patches" - actually will be used to produce sphere of equivalent volume)

cellradius<-2589#nanometers

vacuoleradius<-871#nanometers

vacuolecenter<-c(0,1600,0)

nucleusradius<-890#nanometers

nucleuscenter<-c(0,-200,0)

#Note that the 23 actin patches are modeled here as a single sphere with the same volume as 23 actin patches.

targetsite<-c(0,0,cellradius-targetradius*targetnumber^(1/3))

objtarrad2<-(objectradius+targetradius*targetnumber^(1/3))^2

objvacrad2<-(objectradius+vacuoleradius)^2

objnucrad2<-(objectradius+nucleusradius)^2

objcellrad2<-(cellradius-objectradius)^2

#define some variables we'll use, and set to zero.

walkdistance<-vector(mode="numeric",length=numberoftrials)

contacttime<-vector(mode="numeric",length=numberoftrials)

contacttime[]<-lengthoftrials

contacts<-0

cortexbounces<-vector(mode="numeric",length=numberoftrials)

cortexbounces[]<-0

nucleusbounces<-vector(mode="numeric",length=numberoftrials)

nucleusbounces[]<-0

vacuolebounces<-vector(mode="numeric",length=numberoftrials)

vacuolebounces[]<-0

walk<-matrix(nrow=lengthoftrials,ncol=3)

walk[,]<-0

steps<-matrix(nrow=lengthoftrials,ncol=3)

steps[,]<-0

diffuse<-vector(mode="numeric",length=numberoftrials/1000)

diffusion<-0

#define vectors of x, y and z coordinates of step vector, RMS step length=1

xstep<-vector(mode="numeric",length=lengthoftrials)

ystep<-vector(mode="numeric",length=lengthoftrials)

zstep<-vector(mode="numeric",length=lengthoftrials)

#Start simulation runs:

for (trial in 1:numberoftrials)

{

#pick a random start point on the surface of the nucleus.

startradius<-nucleusradius

#Next pick a random point on the unit circle in the x,y plane.

phase1<-runif(1,min=0,max=2*pi)

#Assign values to the vectors for the x, y and z coordinates of the projections on the unit sphere of start positions for each trial in the actual cell.

zstart1<-runif(1,min=-1,max=1)

ystart1<-cos(phase1)*(1-zstart1^2)^0.5

xstart1<-sin(phase1)*(1-zstart1^2)^0.5

#Assign values to the vectors for the x, y and z coordinates of the start positions for each trial in the actual cell.

xstart<-nucleuscenter[1]+startradius*xstart1

ystart<-nucleuscenter[2]+startradius*ystart1

zstart<-nucleuscenter[3]+startradius*zstart1

#"walk" is a matrix with x,y,z coordinates for each 1 ms interval in the trial

walk[1,]<-c(xstart, ystart, zstart)

#"steps" is a matrix of random movement vectors per step, with lengths normally distributed around either zero, and SD chosen to yield the target diffusion coefficient:

#distance of step

stepradius<-rnorm(lengthoftrials,mean=0,sd=stepsize)

#angular direction of step on z,y plane

phase1<-runif(lengthoftrials,min=0,max=2*pi)

#assign values to vectors of x, y and z coordinates on unit sphere of step vector

xstep<-runif(lengthoftrials,min=-1,max=1)

ystep<-cos(phase1)*(1-xstep^2)^0.5

zstep<-(sin(phase1)*(1-xstep^2)^0.5)

#multiply step radius by x, y and z coordinates on unit sphere of step vector

xstep<-stepradius*xstep

ystep<-stepradius*ystep

zstep<-stepradius*zstep

#"steps" is a matrix of random steps of RMS distance equal to "stepsize".

steps<-cbind(xstep,ystep,zstep)

#assign values for x,y,z coordinates at each millisecond in trial.

for (step in 2:lengthoftrials)

{

#Constant bias in trajectory toward target.

targetdistance<-((walk[step-1,1]-targetsite[1])^2+(walk[step-1,2]-targetsite[2])^2+(walk[step-1,3]-targetsite[3])^2)^0.5

if (runif(1,min=0,max=1000)<stepspersecond) walk[step,]<-walk[step-1,]+steps[step,]+(targetsite-walk[step-1,])*motorstep/targetdistance else walk[step,]<-walk[step-1,]+steps[step,]

#if particle would step past cortex, then pick another element of steps at random and try again - i.e. bounce off cortex, and count bounces.

while(walk[step,1]^2+walk[step,2]^2+walk[step,3]^2>objcellrad2) {walk[step,]<-walk[step-1,]+steps[trunc(runif(1,min=1,max=lengthoftrials)),]

cortexbounces[trial]<-cortexbounces[trial]+1}

#if particle would step into vacuole, then pick another element of steps at random and try again - i.e. bounce off vacuole, and count bounces.

while((walk[step,1]-vacuolecenter[1])^2+(walk[step,2]-vacuolecenter[2])^2+(walk[step,3]-vacuolecenter[3])^2<objvacrad2) {walk[step,]<-walk[step-1,]+steps[trunc(runif(1,min=1,max=lengthoftrials)),]

vacuolebounces[trial]<-vacuolebounces[trial]+1}

#if particle would step into nucleus, then pick another element of steps at random and try again - i.e. bounce off nucleus, and count bounces.

while((walk[step,1]-nucleuscenter[1])^2+(walk[step,2]-nucleuscenter[2])^2+(walk[step,3]-nucleuscenter[3])^2<objnucrad2) {walk[step,]<-walk[step-1,]+steps[trunc(runif(1,min=1,max=lengthoftrials)),]

nucleusbounces[trial]<-nucleusbounces[trial]+1}

#if particle reaches boundary of target, count as contact, record time to contact ("contacttime") and end trial.

if((walk[step,1]-targetsite[1])^2+(walk[step,2]-targetsite[2])^2+(walk[step,3]-targetsite[3])^2<objtarrad2) {contacttime[trial]<-step

contacts<-contacts+1

break

}

}

}

mean(contacttime/1000)

contacttime

#Calculate the average number of minutes a particle takes to reach the target from a random site in the cell.

round(sum(contacttime)/(contacts*60000))

cortexbounces

vacuolebounces

nucleusbounces

for (i in 1:trunc((contacttime[trial]/1000-2)))

{diffuse[i]<-((walk[(i*1000+1001),1]-walk[(i*1000+1),1])^2+(walk[(i*1000+1001),2]-walk[(i*1000+1),2])^2+(walk[(i*1000+1001),3]-walk[(i*1000+1),3])^2)}

mean(diffuse)

palette(rainbow(40))

quartz()

#Plot the projection along the z-axis of the trajectory of the last trial, encoding z-axis position with rainbow color scale.

plot(walk[1:contacttime[trial],1],walk[1:contacttime[trial],2],xlim=c(-cellradius, cellradius),ylim=c(-cellradius, cellradius),col=trunc((walk[1:contacttime[trial],3]+cellradius))/140,pch=19,cex=0.02, main=c(paste("Step size:",motorstep,"; Steps per second:",stepspersecond),"Track color represents Z-coordinate"),xlab="X-coordinate",ylab="Y-coordinate")

quartz()

#Plot the projection along the z-axis of the trajectory of the last trial, encoding fraction of total trajectory duration completed with rainbow color scale.

plot(walk[1:contacttime[trial],1],walk[1:contacttime[trial],2],xlim=c(-cellradius, cellradius),ylim=c(-cellradius, cellradius),col=trunc((1:contacttime[trial])/(0.029*contacttime[trial])),pch=19,cex= 0.02,main=c(paste("Step size:",motorstep,"; Steps per second:",stepspersecond),"Track color represents time"),xlab="X-coordinate",ylab="Y-coordinate")

quartz()

#Plot the projection along the y-axis of the trajectory of the last trial, encoding fraction of total trajectory duration completed with rainbow color scale.

plot(walk[1:contacttime[trial],1],walk[1:contacttime[trial],3],xlim=c(-cellradius, cellradius),ylim=c(-cellradius, cellradius),col=trunc((1:contacttime[trial])/(0.029*contacttime[trial])),pch=19,cex= 0.02,main=c(paste("Step size:",motorstep,"; Steps per second:",stepspersecond),"Track color represents time"),xlab="X-coordinate",ylab="Z-coordinate")
